# A comprehensive retrospect on the current perspectives and future prospects of pneumoconiosis

**DOI:** 10.3389/fpubh.2024.1435840

**Published:** 2025-01-10

**Authors:** Xiaomin Hou, Zhengqian Wei, Xuelu Jiang, Chengjie Wei, Lin Dong, Yanhua Li, Ruifeng Liang, Jisheng Nie, Yiwei Shi, Xiaojiang Qin

**Affiliations:** ^1^Department of Pharmacology, Shanxi Medical University, Taiyuan, Shanxi, China; ^2^Environmental Exposures Vascular Disease Institute, Shanxi Medical University, Taiyuan, Shanxi, China; ^3^China Key Laboratory of Cellular Physiology, Shanxi Medical University, Taiyuan, Shanxi, China; ^4^Department of General Medicine, Second Hospital of Shanxi Medical University, Taiyuan, Shanxi, China; ^5^Academy of Medical Science, Shanxi Medical University, Taiyuan, Shanxi, China; ^6^Department of Foreign Languages, Shanxi Medical University, Taiyuan, Shanxi, China; ^7^School of Public Health, Shanxi Medical University, Taiyuan, Shanxi, China; ^8^Key Laboratory of Coal Environmental Pathogenicity and Prevention, Ministry of Education, Taiyuan, Shanxi, China; ^9^Department of Pulmonary and Critical Care Medicine, First Hospital of Shanxi Medical University, Taiyuan, Shanxi, China; ^10^NHC Key Laboratory of Pneumoconiosis, Department of Pulmonary and Critical Care Medicine, The First Hospital of Shanxi Medical University, Taiyuan, Shanxi, China

**Keywords:** pneumoconiosis, inflammation, fibrosis, current status, cell therapy, nanotechnology

## Abstract

Pneumoconiosis is a widespread occupational pulmonary disease caused by inhalation and retention of dust particles in the lungs, is characterized by chronic pulmonary inflammation and progressive fibrosis, potentially leading to respiratory and/or heart failure. Workers exposed to dust, such as coal miners, foundry workers, and construction workers, are at risk of pneumoconiosis. This review synthesizes the international and national classifications, epidemiological characteristics, strategies for prevention, clinical manifestations, diagnosis, pathogenesis, and treatment of pneumoconiosis. Current research on the pathogenesis of pneumoconiosis focuses on the influence of autophagy, apoptosis, and pyroptosis on the progression of the disease. In addition, factors such as lipopolysaccharide and nicotine have been found to play crucial roles in the development of pneumoconiosis. This review provides a comprehensive summary of the most fundamental achievements in the treatment of pneumoconiosis with the purpose of indicating the future direction of its treatment and control. New technologies of integrative omics, artificial intelligence, systemic administration of mesenchymal stromal cells have proved useful in solving the conundrum of pneumoconiosis. These directional studies will provide novel therapeutic targets for the treatment of pneumoconiosis.

## 1 Introduction

Pneumoconiosis is an incurable but preventable interstitial lung disease caused by occupational inhalation and retention of dust particles (1), such as silica dust, coal mine dust, and asbestos fibers (2). The definition of pneumoconiosis varies from country to country. National Institute for Occupational Safety and Health of USA defines pneumoconiosis as a group of lung diseases caused by the lung's reaction to inhalation of certain dusts (3), and the main cause of the pneumoconiosis is work-place exposure and environmental exposures have rarely been related to these diseases (4). This definition underscores the connection between pulmonary reaction and work place. Pneumoconiosis in the UK is defined as a pulmonary disease caused by inhalation of dust and its retention in the lungs. In China, pneumoconiosis is defined as a disease mainly characterized by diffuse fibrosis of lung tissue caused by long term inhalation of productive mineral dust and their retention in the lungs during occupational activities. Unlike two definitions in the USA and UK, this definition in China focuses on the mineral dust and pulmonary fibrosis.

The emergence of new materials and new industries, such as denim jean production, domestic benchtop fabrication, and jewelry polishing, has resulted in a rising trend of pneumoconiosis, underscoring the need to be cautious about identifying and controlling the sources of novel occupational exposure (5). Although pneumoconiosis is preventable, its incubation period is long and clinical symptoms are not obvious in the early stage. In addition, it could be very difficult to distinguish pneumoconiosis from other lung diseases, including lung cancer, tuberculosis, and sarcoidosis, due to its prolonged latency and similar clinical symptoms (6). Because no effective treatment methods have been established (2), early diagnosis and timely intervention are vital to patients with pneumoconiosis. Therefore, it is of much significance to develop and apply novel diagnostic biomarkers, methods, and therapeutic targets.

This review article aims to provide a more in-depth analysis of the specific pathogenesis, novel diagnostic biomarkers and technologies, new therapeutic targets, prospective drugs, and promising treatments of pneumoconiosis that may be applied on a large scale in the future.

The paper begins with the international and national classifications and epidemiological characteristics of pneumoconioses. Then we broaden the understanding on the strategies for prevention, clinical manifestations, and diagnosis. In the diagnosis of pneumoconiosis, we respectively describe current status of pneumoconiosis diagnosis, prospective biomarkers for diagnosis, and advanced diagnostic techniques. Moreover, to uncover this disease's pathogenesis, autophagy, apoptosis, pyroptosis, and epigenetics on the progression of pneumoconiosis are expressly analyzed. Finally, therapeutic approaches to pneumoconiosis are presented for future investigations, which include the promising targets, promising drugs and other therapies for pneumoconiosis.

## 2 Methodology

Articles were therefore reviewed by searching PubMed, Scopus, Web of Science, Web of Knowledge, Medline, and Google Scholar. The following key words were retrieved: “pneumoconiosis,” “evolution,” “classification,” “prevention,” “manifestation,” “diagnosis,” “pathogenesis,” “inflammation,” “fibrosis,” “therapy,” “treatment,” etc.

## 3 International and national classifications of pneumoconioses

Specific kinds of pneumoconioses are listed as occupational diseases by International Labor Organization (ILO) and Chinese government. Based on *The Occupational Diseases Catalogue, 2010 edition* released by ILO, pneumoconioses can be divided into two types: pneumoconiosis caused by fibrous mineral dust and pneumoconiosis caused by non-fibrous mineral dust (7). In the *Classification and Catalogue of Occupational Diseases, 2013 Edition* released in China, twelve kinds of pneumoconioses have been listed as statutory occupational diseases, including silicosis, coal workers' pneumoconiosis, graphite pneumoconiosis, carbon black pneumoconiosis, asbestosis, talc pneumoconiosis, cement pneumoconiosis, mica pneumoconiosis, kaolin pneumoconiosis, aluminosis, electric welder pneumoconiosis, and foundry worker pneumoconiosis. Since it is sometimes difficult to determine the main cause of pneumoconiosis, people still name the disease according to the patients' occupations, such as welder's pneumoconiosis and grinder's pneumoconiosis.

Apart from above kinds of pneumoconioses listed as occupational diseases, several classification systems of pneumoconioses have been devised over the years on different criteria. The specific classifications of pneumoconioses in some countries and regions are listed in Table 1. According to *International Classification of Diseases, 11*^*th*^ *Edition*, pneumoconioses can be divided into 11 major categories, and there are three subgroups of pneumoconiosis caused by siliceous dust. In Japan, silicosis in miners was not identified as an occupational disease until 1930. Silicosis caused by free silica dust and silicosis with tuberculosis were identified as occupational diseases in 1936 (8). Several other types of pneumoconiosis, such as asbestosis and talc pneumoconiosis, were also added. Based on the recommendation of National Institutes of Health of USA, pneumoconioses can be divided into five types: silicosis, asbestosis, coal workers pneumoconiosis, other kinds of pneumoconiosis, and benign pneumoconiosis (9). European Union classified pneumoconioses into four categories: silicosis, silicosis with tuberculosis, asbestosis, and pneumoconiosis caused by silicate dust. Only three types of pneumoconiosis are identified in India, including silicosis, asbestosis, and coal workers pneumoconiosis (10). In China, pneumoconioses can be divided into five categories based on the type of dust inhaled: silicosis caused by inhalation of dust containing free silica; silicate lung caused by inhalation of dust containing silica such as asbestos, mica, and nephelite; carbon pneumoconiosis caused by inhalation of coal, graphite, activated carbon, carbon black and other powders; mixed pneumoconiosis caused by inhalation of dust containing free silica and other types of dust (organic dust, inorganic dust, synthetic dust); and other pneumoconioses, caused by metal or other compounds, such as aluminum, and glass wool (11).

**Table 1:** International and national classifications of pneumoconioses.

**Nation**	**Number of categories**	**Type**
International level	11	Pneumoconiosis caused by silica dust (CA60.0) Talc pneumoconiosis (CA60.00) Specific pneumoconiosis caused by silica dust (CA60.0Y) Unspecified pneumoconiosis caused by silica dust (CA60.0Z) Coal workers' pneumoconiosis (CA60.1) Pneumoconiosis caused by mineral fibers (CA60.2) Pneumoconiosis related to tuberculosis (CA60.3) Aluminosis (CA60.4) Pulmonary bauxite fibrosis (CA60.5) Berylliosis (CA60.6) Pulmonary graphite fibrosis (CA60.7) Siderosis (CA60.8) Tin pneumoconiosis (CA60.9) Other specific pneumoconioses (CA60.Y)
Japan	8	Silicosis Asbestosis Welder's lung Coal workers pneumoconiosis Aluminum pneumoconiosis Indium pneumoconiosis Beryllium pneumoconiosis Cemented carbide pneumoconiosis
America	5	Silicosis Asbestosis Coal workers' pneumoconiosis Other kinds of pneumoconioses Benign pneumoconiosis
European Union	4	Silicosis Silicosis with tuberculosis Asbestosis Pneumoconiosis caused by silicate dust
China	5	Silicosis caused by inhaling dust containing free silica Silicosis caused by inhaling dust containing silica Carbon pneumoconiosis Mixed pneumoconiosis Other pneumoconioses
India	3	Silicosis Asbestosis Coal workers' pneumoconiosis

## 4 Epidemiological characteristics of pneumoconiosis

According to the *Global Burden of Disease Study 2019*, a total of 0.20 (0.17–0.23) million new cases of pneumoconiosis were diagnosed and 0.92 (0.76–1.12) million disability-adjusted life years (DALYs) were calculated in the year 2019, implying that pneumoconiosis is still a major concern worldwide (12). Based on the *Global Burden of Disease Study 2017*, cases of pneumoconiosis have increased in the five sociodemographic index regions from 1990 to 2017 while the age-standardized incidence rates (ASIR) have shown a downward trend. A reduction was observed in ASIR of silicosis, coal workers' pneumoconiosis (CWP), and other pneumoconioses. However, the ASIR of asbestosis displayed an increasing trend (13). Increase in ASIR of asbestosis was the biggest in high-income continents like North America and Australasia, suggesting the importance of intensifying the controlment of asbestos in the market. In addition, higher incidence of pneumoconiosis was observed in males than in females. Besides, the highest ASIRs in 2017 were noted in China, Papua New Guinea, and North Korea. Middle- Socio-demographic Index regions had the highest ASIR of pneumoconiosis. The ASIR of pneumoconiosis is inversely correlated with human development index (14).

Distinct occurrence patterns of pneumoconiosis have been reported in different countries and regions (13). For example, in America, pneumoconiosis-associated-deaths decreased by 40.4% from 1999 to 2018. CWP (69.6%) and silicosis (53.0%) accounted for the largest proportion of declining cases. On the other hand, asbestosis was the most reported pneumoconiosis, which was consistent with the world epidemic trends of pneumoconiosis. The matter of concern was that the incidence of pneumoconiosis due to other inorganic productive dust increased dramatically (e.g., aluminum, bauxite, beryllium, iron, and tin oxide) (1). Notably, the national prevalence of CWP among working coal miners is increasing, especially in central Appalachia, which will likely be reflected in future trends for severe and disabling disease, including progressive massive fibrosis (15), and it is speculated that the nano-sized coal dust is likely to be one of the reasons for the increase in the prevalence of CWP (16).

China suffered from the world's largest health loss from pneumoconioses in 2019, accounting for two-thirds of the global health loss from pneumoconiosis (2). According to the *Occupational Diseases Report in 2021*, 11,809 newly reported cases of occupational pneumoconiosis were diagnosed in China, accounting for 77.65% of all new occupational disease cases, making it urgent to prevent and control pneumoconiosis. Newly diagnosed cases and DALYs of pneumoconiosis continued to rise during 1990–2019 in China. However, age standardized calculation showed a significant downward trend in the incidence, death, and DALY rates due to pneumoconiosis, with the exception of incidence of silicosis and asbestosis-associated mortality (2). This achievement was mainly attributed to the unremitting efforts of Chinese government. Other factors which contributed to this decline include intensified regulatory supervision, expanded in medical accessibility, and improved medical treatment. In China, silicosis accounted for the largest proportion of confirmed pneumoconiosis cases, followed by CWP, other pneumoconiosis, and asbestosis in 2019 (17). Notably, the age-standardized death rate of pneumoconiosis in western China was higher than that in the eastern coastal area of China, and there is an urgent need for adequate supervision and medical services of occupational diseases in Western China (2). Additionally, new cases, deaths, and DALYs due to pneumoconiosis in males accounted for approximately 95% of the corresponding total numbers in 2019, which was consistent with previous reports (2).

Accurate data on the prevalence of pneumoconiosis is essential for health resource planning and policy development. In 2016, pneumoconiosis was found to have caused 21,488 deaths worldwide (13). However, it must be pointed out that the actual burden of pneumoconiosis in China may be greater than reported, which could be caused by the following factors. First, due to the low frequency of occupational health examinations and absence of strict diagnostic criteria for pneumoconiosis (18), timely diagnosis is not available for all potential patients in relevant industries, leading to an underestimate of existing cases. Secondly, workers in small industrial enterprises or small informal workshops tend to solve health and safety problems by themselves, and such cases go unreported (19). Thirdly, the frequent flow of migrant workers and long incubation period of pneumoconiosis can also result in underreporting (2). Besides, some workers are reluctant to take a physical examination, especially a chest examination for fear of losing jobs, even if relevant symptoms have appeared (20).

With the emergence of new industries, such as denim jean production, benchtop fabrication, and jewelry polishing (5), the incidence of pneumoconiosis, especially silicosis, is increasing. Workers may inhale fumes containing sand and other toxic chemicals while sandblasting, which is used to get the look of buff pants (21). The incidence of silicosis is on the rise in Spain, Australia, and some other regions where artificial stones (AS) get popular (6). Unlike natural stone associated silicosis, AS-associated silicosis was characterized by short latency, rapid radiological progression, accelerated decline in lung function, and high mortality (17). The respirable crystalline silica (RCS) is a by-product of AS production, and the dust is composed of inhalable particles with a diameter usually smaller than 5 μm (6). RCS could be disregarded easily because it is colorless, odorless, and accumulates quickly. Much exposure to RCS has been reported to result in the occurrence and progression of silicosis (6). Likewise, increase in the use of nanomaterials due to the emergence of nanotechnology, in which the generated nanoparticles are cytotoxic to lung epithelial cells, has resulted in a higher incidence of pulmonary inflammation and fibrosis (22).

## 5 Strategies for preventing pneumoconiosis

Although pneumoconiosis poses a major threat to global public health, preventive measures can be taken to reduce its harm. Medical workers need to perform follow-up visits for key populations, including patients diagnosed with advanced pneumoconiosis at first diagnosis, patients of older age, and patients with prolonged exposure to dust, and strict supervision should be carried out in industries with high incidence of pneumoconiosis, such as coal mining and construction (23).

Workers at risk of pneumoconiosis need occupational safety education to raise their awareness of personal protection, ensuring that they are responsible for their health (1). Rapid progression of pneumoconiosis in miners has been reported to be strongly correlated with exposure to high concentrations of mineral dusts (24), indicating the necessity of clean working environment.

Employers and business enterprises should ensure that sufficient personal protective equipment forms an indispensable part of the workers' gear. Workers can use filter type dustproof respiratory protective equipment to prevent the inhalation of dust in the working environment. Protective efficiency of filtering dust respirators relies on the properties of filter materials, from which filters are made, and the structure of a half mask frame. A new half mask frame design has been proposed, which is flexible and fits the face surface well (25). The priority is to improve the controlling measures, including elimination, substitution, and exhaust ventilation, in addition to the supply of respirators (6). It is important to use technical measures of ventilation, dust removal, detoxification, noise reduction, and isolation, to eliminate dust hazards. Wet dedusting is the main coal dust suppression technique commonly used in coal mines, and coal wettability is the main factor that influences dust suppression efficiency (26). Based on the two-fluid (Euler-Euler) frame model, a mathematical model for wet deducting process has been established to explore the effect of particle size distribution of the dust particle, spray flow, and ventilation rate on the dedusting efficiency of wet dedusting method. This model revealed that droplets with a diameter between 15 μm and 70 μm can ensure high capture efficiency of respirable dust, and that spray quantity and dedusting efficiency are not necessarily proportional (27). Another mathematical model was proposed for cyclonic spray dedusting, and the spray can be used to promote agglomeration of particles and improve the dust removal efficiency in the swirl field (28). With the increased attention toward pneumoconiosis and rapid development of science and technology, several novel wet dedusting technologies have been invented, including pneumatic spiral spray system (29), novel wind-assisted centralized spraying dedusting device (30), and pre-injection foam dedusting technology (31). With remarkable dust suppression performance, these technologies can help to improve the work environment effectively. Furthermore, employers should provide some medical services for workers in the forms of health questionnaires, physical examination, lung function, and chest radiology (6). Moreover, both employers and health care providers should inform relevant public health agencies of the identification of cases, so that timely interventions can be performed and adequate treatment can be administered to patients with pneumoconiosis (23, 32).

Government should improve surveillance and guarantee systems of occupational diseases, step up the intensity of regulatory surveillance, and instruct employers to standardize the protocols of safe production (1). It is also necessary to increase the investment in frontier research and technology development to explore novel measures of prevention. Meanwhile, the government should encourage the use of replaceable and harmless productive materials to reduce the incidence of pneumoconiosis (2). A systematic review indicates that exposed workers suffer from a higher risk of lung cancer when asbestosis or silicosis is present (33). Attention should also be paid to pneumoconioses caused by other inorganic dust, for the incidence rate is rising in recent years. Inhalation of silica dust is correlated with systemic autoimmune diseases (34), and it is important to take an occupational history in patients with autoimmune diseases to improve recognition of workplace silica exposure (32).

## 6 Clinical manifestations and diagnosis of pneumoconiosis

### 6.1 Clinical manifestations

**Figure 1:**
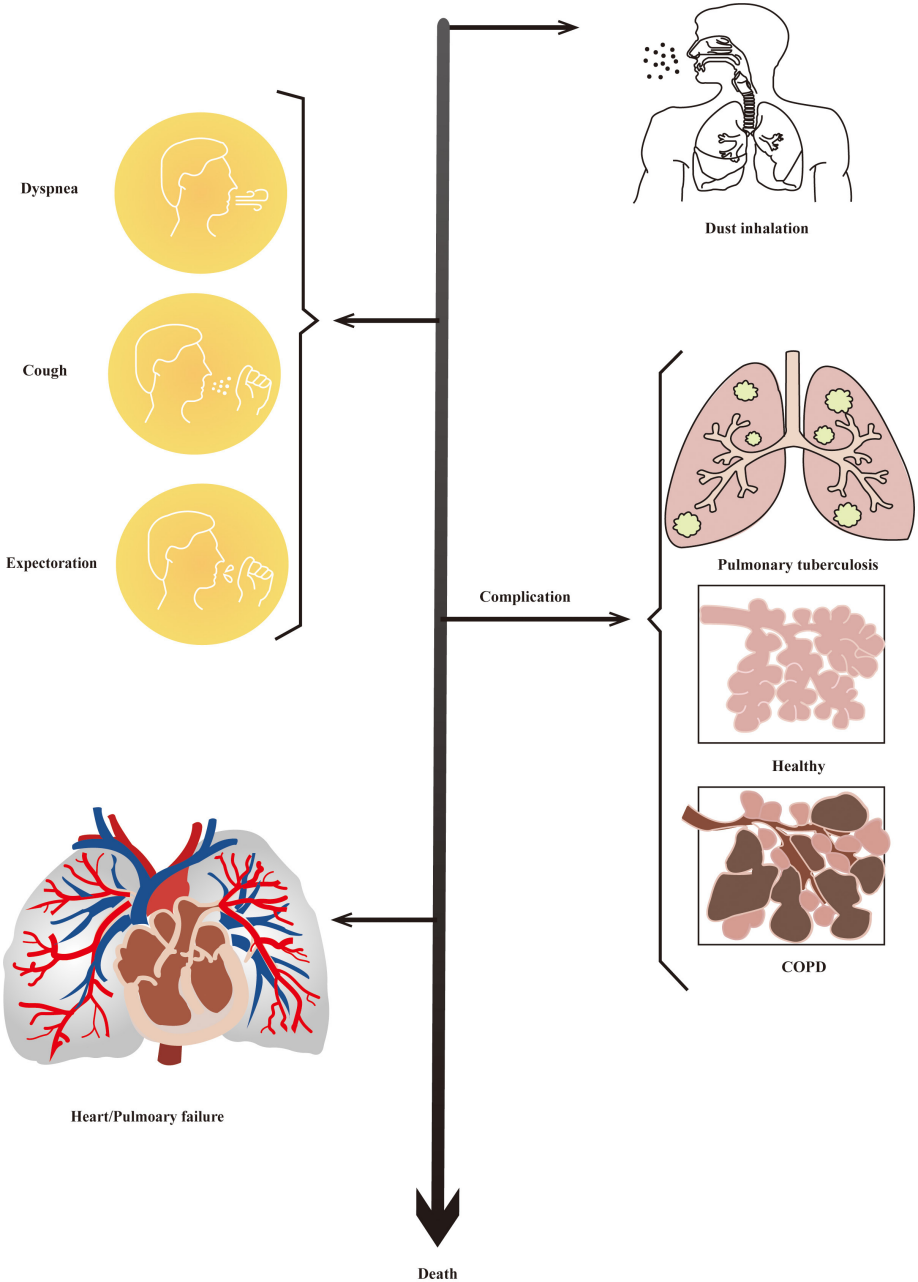
Clinical manifestation of pneumoconiosis. Most people have no obvious symptoms in the early stages of pneumoconiosis. Then, dyspnea, cough, expectoration, and chest pain would be the main manifestation of pneumoconiosis. There would be a series ofcomplications including tuberculosis, emphysema, and COPD at the irreversible stage of progression. Finally, patients would die of respiratory and/or heart failure.

The incubation period of pneumoconiosis is long, and the majority of the patients do not show any obvious symptoms. The main clinical manifestations of pneumoconiosis are dyspnea, cough, expectoration, and chest pain (23), which appear after persistent exposure to mineral dusts. Most of patients are often in the irreversible stage with a series of complications, including tuberculosis, emphysema, and chronic obstructive pulmonary disease (COPD) (2, 35). Pulmonary inflammation and progressive fibrosis are typical pathological changes of pneumoconiosis, which can result in respiratory and/or heart failure (36). Clinical manifestation and the developing process of pneumoconiosis is shown in Figure 1.

### 6.2 Diagnosis of pneumoconiosis

#### 6.2.1 Current status of pneumoconiosis diagnosis

Pneumoconiosis is diagnosed on the basis of functional changes of lungs identified by pulmonary function test (PFT) and morphological changes of lungs determined by radiological findings, including computed tomography (CT) scanning of the chest, chest radiography, or lung biopsy findings in conjunction with occupational history exposure to mineral dust, clinical manifestations, and working environment (23, 32, 37, 38). PFT shows that patients have normal ventilation function in early stage of pneumoconiosis, and their dispersion function decreases during middle and late stages with varying degrees of restrictive or obstructive ventilation disorders. Besides, auxiliary examinations also include X-rays and arterial blood gas analysis. Arterial blood gas analysis shows patients of pneumoconiosis suffer from hypoxemia, and hypercapnia aggravates the situation in advanced stage. As common characteristics of pneumoconiosis, Pulmonary inflammation, and fibrosis can be used as diagnostic clues (6).

It indicates the possibility of pneumoconiosis if there are presence of nodular or reticulonodular lesions in chest radiography or small nodules with peri lymphatic distribution on thin-section CT with or without eggshell calcifications in the population exposed to dust (39). Current diagnostic criteria are based on the *International Labour Organization*/*International Classification of Radiograph of Pneumoconiosis (ILO/ICRP), 2011 Edition*, which contains a set of digital standard images. The X-ray images of pneumoconiosis are characterized by small opacities and pleural plaques, which are not specific to this disease (23). ILO has subdivided pneumoconiosis from three aspects: technical quality, substantial abnormalities, and pleural abnormalities. According to the guidelines of ILO/ICRP, parenchymal abnormalities of pneumoconiosis can be divided into small opacities and large opacities. Small opacities have three subdivisions in profusion, shape and size, and mixed. Large opacities have four levels of 0, A, B, and C. Based on the *GBZ70-2002-Diagnostic Criteria for Pneumoconiosis*, China divides the progression of pneumoconiosis into three stages, and the radiographic criteria include the overall density of small opacities, lung range of small opacities, and whether small opacities converge into big opacities, pleural plaques or other symptoms, China divides pneumoconiosis into three stages based on the GBZ70-2002-diagnostic criteria for pneumoconiosis. Specific diagnostic criteria for each stage are listed in Table 2.

**Table 2:** The diagnostic stages of pneumoconiosis in China.

**Diagnostic stages**	**Reference symptoms**
Stage I	a) There is a small opacity with an overall density of 1, which is distributed in at least 2 lung regions. b) Exposure to asbestos dust showed a small opacity with an overall density of 1, distributed in only 1 lung area, and pleural plaques appeared. c) Exposure to asbestos dust resulted in an overall concentration of small opacities of 0, but a concentration of small opacities of 0/1 in at least two lung areas accompanied by pleural plaques (People with one of these symptoms can be diagnosed with pneumoconiosis.)
Stage II	a) There is a small opacity with an overall density of level 2, distributed over 4 lung regions. b) There was a small opacity with an overall density of level 3, and the distribution range reached 4 lung regions. c) Exposure to asbestos dust, a small opacity with an overall density of 1, distributed in more than 4 lung areas, with pleural plaques that have involved part of the heart margin or diaphragmatic surface. d) Exposure to asbestos dust, with a small opacity of overall concentration level 2, distributed over 4 lung areas. Pleural plaques are present at the same time and have involved part of the heart margin or diaphragmatic surface. (People with one of these symptoms can be diagnosed with pneumoconiosis.)
Stage III	a) There is a large opacity with a long diameter of no < 20 mm and a short diameter of no < 10 mm. b) There are small opacities with an overall density of level 3 distributed over 4 lung regions with small shadow clusters. c) There is a small opacity of total intensity level 3 and the distribution range is more than 4 lung regions with large shadow. d) Exposure to asbestos dust presents a small opacity with an overall concentration of grade 3, covering more than 4 lung regions, and the length of a single or multiple pleural spots on both sides exceeds half of the length of the unilateral chest wall or involves the heart margin and makes it partially disheveled. (People with one of these symptoms can be diagnosed with pneumoconiosis.)

Notably, it is not easy to distinguish pneumoconiosis from lung cancer, tuberculosis and sarcoidosis due to prolonged latency and similar clinical symptoms (6). With the rapid development of technologies, magnetic resonance imaging has proven useful in distinguishing progressive massive fibrosis from lung cancer (39). Bronchoalveolar lavage fluid can also be used to detect the biomarkers of pneumoconiosis, which will be of much helping accurate diagnosis.

#### 6.2.2 Prospective biomarkers in pneumoconiosis diagnosis

Early and accurate diagnosis of pneumoconiosis is difficult due to the delayed appearance of clinical manifestations and the complexity of the diagnostic procedures, and this is why the majority of the patients are already in the advanced stage at the time of diagnosis, when the condition is irreversible and no effective treatment is available. Fortunately, with the deepening research on the pathogenesis of pneumoconiosis in the recent years, new biomarkers have been discovered and can be potentially used as novel diagnostic tools. Prospective biomarkers of pneumoconiosis in the process of diagnosis are summurized in Table 3.

**Table 3:** The biomarker of pneumoconiosis.

**Biomarker**	**Expression level**	**Tissue/cell/patients**	**References**
HO-1	Up	Blood serum	(40)
PGD	Up	Human lung tissue	(41)
Thromboxane A (TXA)	Up	Human lung tissue	(41)
IL-8	Up	Blood serum	(42)
Tumor necrosis factor α (TNF-α)	Up	Blood serum	(42)
HECTD1	Up	AMs and RAW264.7 macrophage cell line	(44)
High-mobility group box-1 (HMGB-1)	Up	Blood serum	(53)
Apoptosis of AMs	Up	Patients with silicosis	(46)
circulating double-stranded DNA	Up	Sputum	(54)
C-X-C motif chemokine ligand 10	Up	Sputum	(54)
Krebs von den Lungen 6	Up	Blood serum	(55)
Surfactant protein-D	Up	Blood serum	(55)
Matrix metalloproteinase-2	Up	Blood serum	(55)
Npnt	Up	Patients with silicosis	(45)
Activating transcription factor 3	Up	Patients with silicosis	(43)
Propylparaben	Up	Blood serum	(56)
Has-miR-4516	Up	Blood serum	(48)
Circular RNA hsa_circ_0058493	Up	Peripheral blood	(57)

A previous study on patients with silicosis and BALB/c mice shows that increasing the content of pulmonary heme oxygenase-1 (HO-1) can inhibit the activity of reactive oxygen species (ROS) and subsequent pathologic changes, thereby attenuating progression of silicosis (40). Cytokines and inflammatory factors, as well as proteins, can be helpful in the diagnosis and treatment of pneumoconiosis. Some cytokines and inflammatory factors, such as interleukin (IL-8) and Prostaglandin D (PGD), can be used as indicators of pneumoconiosis (41, 42). Transcription factor 3, an inflammatory repressor, can be activated for early diagnosis of silicosis (43). In addition, changes in the expression of some proteins can also be used for the diagnosis of pneumoconiosis. For example, HECT domain E3 ubiquitin ligase 1 (HECTD1) may serve as a potential marker of silicosis, as it promotes silica-induced activation of macrophages via ubiquitination, thereby inducing proliferation and migration of fibroblasts (44). Similarly, development of fibrosis can be recognized by serum nephronectin (Npnt), which is a new member of the integrin family of ligands, suggesting that Npnt seems to play a role in the progression of fibrosis with other cytokines and can be used in the diagnosis of pneumoconiosis (45). Alveolar macrophages (AMs) apoptosis could be used as a potential biomarker for human silicosis, which promotes the development and progression of silicosis via activating the fatty acid synthetase (Fas)/fatty acid synthetase ligand (FasL) pathway (46).

MicroRNAs (miRNA) have great application prospects as biomarkers in diagnosing pneumoconiosis, and they may serve as indicators of organ or cell-specific toxicity, disease, and biological status (47). It is noteworthy that has-miR-4516 targeted genes encodes basonuclin2, inhibitors of growth family member 4, the potassium voltage-gated channel, and “sha-1-related subfamily member 1” proteins, which shows that has-miR-4516 could be used as a potential biomarker of pulmonary fibrosis progression in patients with pneumoconiosis (48). Besides, an increase was observed in miR-107 in serum exosomes and lung tissue in the experimental silicosis mouse model, while the inhibition of miR-107 reduced pulmonary fibrosis, which provided a rationale for using miR-107 for intervening in silicosis progression (49). However, miRNAs have not been routinely used as non-invasive biomarkers, for lack of standard approaches to sample preparation and miRNA measurement, as well as uncertainty in their biological interpretation (50).

Circular RNAs (circRNAs) are non-coding RNAs with a closed loop structure, and they are identified as competing endogenous RNAs (ceRNAs) serving as a sponge for miRNA through complementary base paring (44). Long non-coding RNAs (IncRNAs) are a large class of non-coding transcripts of >200 in length with no protein-coding capacity, and they are involved in chromosome modification, transcription and post-transcriptional processing (51). Several studies have demonstrated that IncRNAs can function as ceRNAs in the process of fibrosis by binding to and undergoing crosstalk with miRNAs, and more relevant research can be conducted on IncRNAs (52).

#### 6.2.3 Advanced diagnostic techniques for pneumoconiosis

With the rapid development of science and technology, more and more diagnostic methods have been developed for the diagnosis and assessment of severity of silicosis, and high-resolution computed tomography (HRCT) and chest radiography (CR) are two of them. A previous study shows that HRCT is more reproducible and more accurate than CR in the diagnosis of early pneumoconiosis, suggesting that HRCT is more correlated with lung function test. However, the results of the study did not support the hypothesis that HRCT was more sensitive than CR in the early detection of silicosis (58). Electrical impedance tomography (EIT) can be used to detect the spatial distribution of electrical properties of tissues by measuring the transfer impedances between electrodes on the body surface (59). This technique is usually applied to functional chest examinations with the purpose of identifying patients with chronic pulmonary diseases at early stage (60). The feasibility of EIT perfusion imaging has been proved (61). Although EIT has widespread applications, uniform diagnostic criteria have not been developed, and recommendations are needed on how EIT findings can be used to generate diagnoses. Magneto pneumography was invented to investigate the remanent magnetism of foreign intrathoracic ferromagnetic particles after magnetization by an external magnetic field, and one advantage of this technique is noninvasiveness (62). A lot of work has to be done in this field before it can be widely used in clinic practice, due to its uncertainty in safety, sensitivity, and specificity (37).

Artificial Intelligence (AI) has several development prospects in the diagnosis and management of pulmonary diseases (63), such as lung nodule evaluation, tuberculosis or pneumonia detection, and quantification of diffuse lung diseases (64). Computer-aided detection based on machine learning is an emerging research field, especially artificial neural network and convolutional neural networks (64, 65), which have demonstrated significant performance gain over the classic machine learning techniques. AI-assisted radiography screening and diagnosis in occupational lung diseases has proven feasible and effective (66). CR is a near perfect domain for the development of deep learning algorithms for automatic interpretation, requiring large annotated datasets, in view of the high number of procedures and increasing data availability (64). Compared with classic machine learning techniques, deep learning methods have led to substantial performance gain (64). Zhang et al. set out to establish an AI-based model, which could help doctors to diagnose pneumoconiosis and stage the course of the disease through CR. The system of chest X-ray was created with the help of a training queue and confirmed with the help of an independent evaluation queue. Their groundbreaking study evaluated the possibility and effectiveness of AI-assisted radiological diagnosis and screening in the field of occupational lung disease (66). Zhang et al. proposed a technique of diagnosing pneumoconiosis using wrist pulse signals, in which wrist pulse signals were collected from both non-pneumoconiosis and pneumoconiosis patients, and then a single piece of pulse signal was separated. They used machine learning methods to process and analyze the pulses, and reported that their 13-dimensional feature could be used as the main feature for the diagnosis of pneumoconiosis (67).

## 7 Pathogenesis of pneumoconiosis

The pathogenesis of pneumoconiosis involves several complex mechanisms. In this review article, silicosis, the most common type of pneumoconiosis, has been taken as an example to elucidate the pathogenesis of pneumoconiosis.

### 7.1 Basic mechanism of silicosis

Silica particles entering the distal airways and alveoli have to be RCS, with a diameter usually smaller than 5 μm (6). When these particles arrive at their destination, the alveolar macrophages (AMs) identify and swallow silica dusts through macrophage receptor with collagenous structure (MARCO) (68, 69). Silica (Si-) and Silicon monoxide (SiO-) radicals react with water to generate ROS, reactive nitrogen species, and nitic oxide (NO), which could lead to lipid peroxidation of cell membranes and apoptosis in macrophages and other cells (70). Macrophage polarization is closely associated with the occurrence and development of silicosis, and could be the key to further elucidating the pathogenesis of silicosis (71). Classically activated macrophage is the primary type of alveolar macrophage polarization occurring in the early stage of silicosis. As the disease progresses, alternatively activated macrophage gradually becomes the dominant type of polarization to promote tissue repair. Additionally, signal transducer and activator of transcription (STAT) and interferon regulatory factor signaling pathways are also involved in the process of macrophage polarization in silicosis (71). Moreover, the activation of nucleotide-binding oligomerization domain-receptor interacting protein 2-nuclear factor-k-gene binding signaling pathway may also lead to the polarization of macrophages (72). Besides, the cyclic GMP-AMP synthase (cGAS) activates the stimulator of interferon genes (STING) and then brings about an increase in ROS generation. The cGAS-STING pathway, as a result, is crucial for silica-induced pulmonary inflammation (73). When silica particles are phagocytosed, the H-bonding reaction occurs and damages the lysosomes in AMs. Lysed lysosomes activate the inflammasome, a polyprotein complex in the cytoplasm, and the activation leads to the increased expression of inflammatory cytokines, including TNF-α, transforming growth factor β (TGF-β), and interleukin 1β (IL-1β). A previous study demonstrated a potential role of IL-1beta-dependent NO-mediated apoptosis in evolution of murine silicosis, thereby showing an association between apoptosis and inflammation (74).

**Figure 2:**
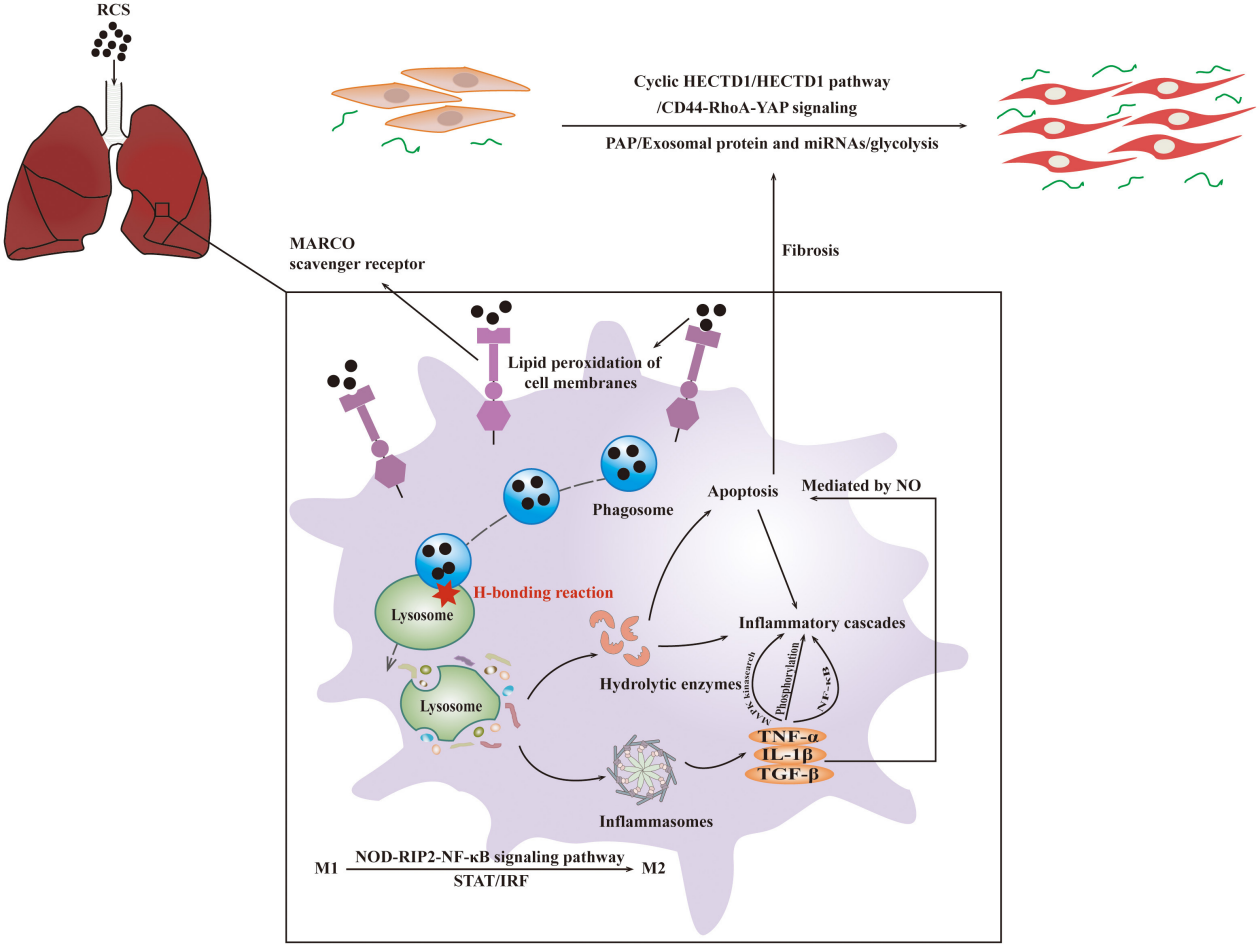
The basic mechanism of silicosis. RCS particles inhaled would be identified and swallowed by MARCO of AMs. After entering the cytoplasm, crystalline silica would cause the lysosome to break, resulting in the activation of inflammasome followed by the inflammatory cascades and fibrosis. The interaction of these mechanisms will lead to apoptosis.

Periodic acid-Schiff-positive material accumulation occurs in the alveoli due to dysfunction of AMs, and this accumulation is the typical characteristic of pulmonary alveolar proteinosis (PAP) (75). PAP could promote profibrotic response by transforming fibroblasts into myofibroblasts through LOC103691771 induced by TGF-β1 (52), and PAP also would result in collagen deposition and fibrosis of lungs (76). Fibrocytes and myofibroblasts derived from lung type II epithelial cells play a crucial role in the early stage of silicosis, while myofibroblasts derived from resident lung fibroblasts play a key role during the formative period of fibrosis (77). Additionally, the extracellular matrix-related molecules, such as integrins and their ligands including fibronectin, vitronectin, laminin, and collagens, also exert an important influence on the process of fibrosis (45). Activation of fibroblast is initiated by cluster of differentiation 44-ras homolog gene family-yes-associated protein (CD44-RhoA-YAP) signaling (78). A study demonstrated the fibrogenic effect of glycolysis through the circular RNA HIPK3 (Homeodomain-interacting protein kinase)/micro-30a-3p/Forkhead box K2 regulatory pathway, indicating the important role of glycolysis in the development of pneumoconiosis (79). Exosomal protein and miRNAs including sulphation of secreted phosphoprotein I and a miR-125a-5p derived from macrophage exosomes, used for intercellular communication, have been reported to play a key role in fibroblast trans differentiation and the development of silicosis (80, 81). Silica-exposed macrophage-derived exosomes promote the progression of fibrosis, which is mediated by endoplasmic reticulum (ER) stress (82). The basic mechanism of silicosis is presented in Figure 2.

### 7.2 Autophagy on the progression of pneumoconiosis

**Figure 3:**
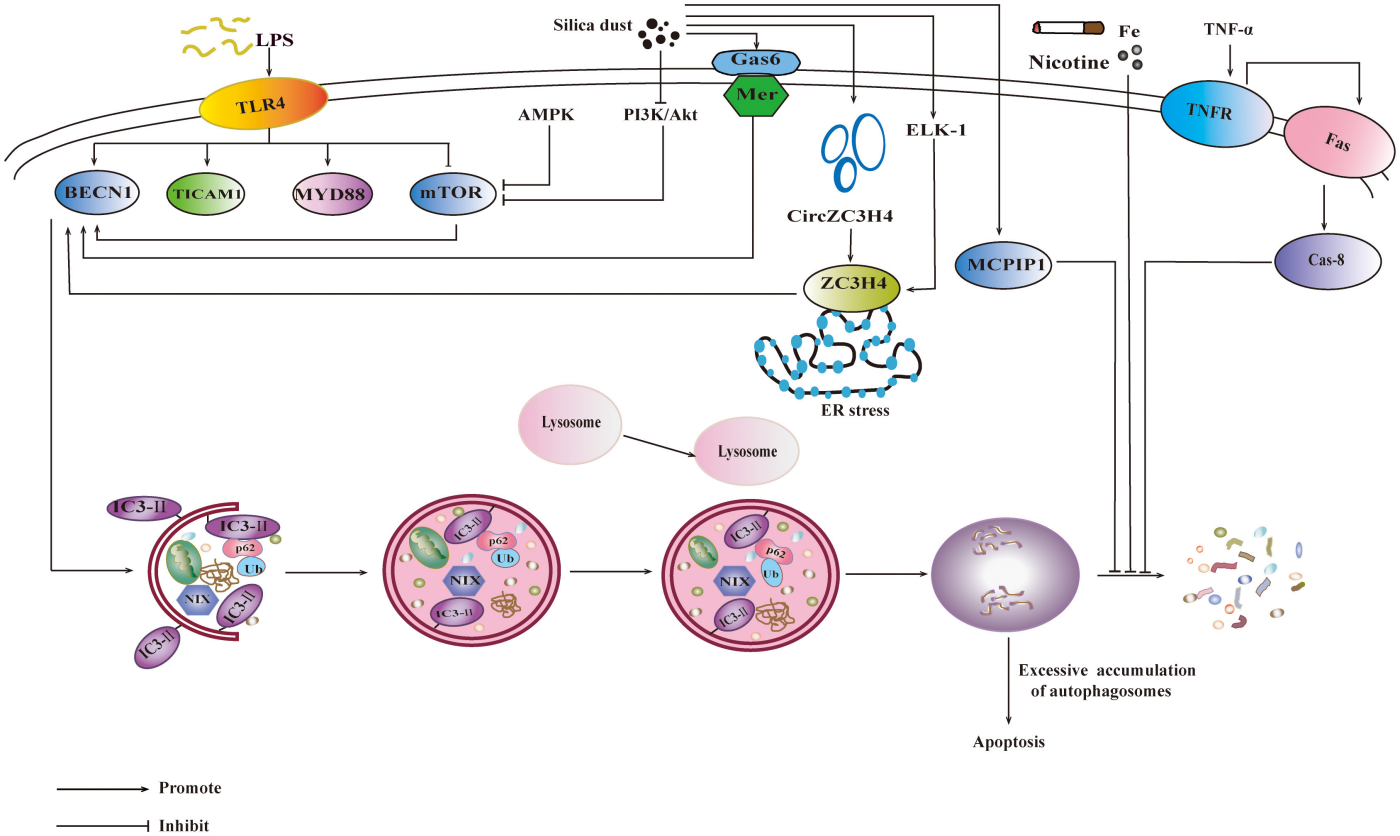
Influence of autophagy on the progression of pneumoconiosis. PI3K/Akt/mTOR signaling pathway and AMPK-mTOR signaling pathway are involved in the silica-induced autophagy. The activation of Gas6/Mer would also promote autophagy. ZC3H4 is involved in autophagy by ER stress, which is promoted by ELK-1. The increase of monocyte chemoattractant protein-induced protein 1 could promote autophagy. FAS- caspase-8 inhibits autophagy through TNF-α-TNFR signal pathway. Environmental factors, including lipopolysaccharide (LPS), nicotine etc., also exert influence on autophagy.

It was reported that phosphatidylinositol3-kinase/Protein Kinase B/Mechanistic Target of Rapamycin (PI3K/Akt/mTOR) signaling pathway was involved in the autophagy induced by silicon dioxide exposed, and autophagy may play a protective role in the process of pulmonary fibrosis (83). Autophagy can also be activated by the adenosine monophosphate activated protein kinase/mammalian target of rapamycin (AMPK-mTOR) signaling pathway (84). Genetic loss of Gas6 reduces the expression of Mer receptor, leading to the decline of accumulation of silica-induced autophagosomes (85). Autophagy plays a two-sided role in the occurrence of silicosis. Under normal circumstances, autophagy degrades intracellular matter to produce new building blocks and energy for cellular renovation and homeostasis (86). With the ability of reducing apoptosis of alveolar epithelial cells, autophagy could relieve silica-induced fibrosis (87). However, inhaling silica particles cause lysosomal rupture leading to excessive accumulation of autophagosomes in AMs, which may lead to apoptosis in AMs (88). ZC3H4, a member of the Cys-Cys-Cys-His (CCCH) zinc finger protein family, is involved in silica-induced endothelial-mesenchymal transition (EndoMT) through ER stress and autophagy (89). ZC3H4 was reported to regulate the secretory function of monocytes, which, in turn, inhibited fibroblast function in early inflammation through autophagy signaling, thereby reducing pulmonary fibrosis (90). ETS-like transcription factor (ELK-1) could promote epithelial mesenchymal transition (EMT) via the upstream activity of OS and downstream signaling of ZC3H4 expression (85). It was reported that silica dust exposure could induce autophagy by changing the connectivity of Beclin1 from Bcl-2 to PIK3C3 (91). Autophagy could be inhibited by FAS- caspase-8 due to the activation of TNF-α-TNF-receptor (TNF-α-TNFR) signal pathway, which results in apoptosis of AM (92). In the progression of pneumoconiosis, signaling pathways and targets involved under the influence of autophagy are shown in Figure 3.

### 7.3 Apoptosis on the progression of pneumoconiosis

Silica-induced apoptosis exerts an inflammatory effect in the lung parenchyma and creates immunologic abnormalities in the regional lymph nodes, which could promote the progression of silicosis (93). Fas/FasL pathway may regulate the process of apoptosis. Expression of Fas ligand was reported to increase after silica inhalation, and led to the apoptosis of Fas ligand-dependent macrophage (94). Cytochrome c can help the combination of apoptotic protease activating factor-1 (APAF-1) and pro-caspase 9, resulting in the enzymatic cascade and apoptosis (95). TNFR1/Phox interaction is a crucial event in the pathogenesis of silicosis, and it inhibits the formation of mitochondrial ROS (mtROS) and reduces macrophage apoptosis (96). Up-regulation of Bax and down-regulation of Bcl-2 lead to the cleavage of caspase-9 and activization of caspase-3, respectively. Caspase-8 could activate caspase-3 through extrinsic apoptin pathway, thereby initiating a caspase-cascade and cell apoptosis (97). p53 plays a significant role in silica-induced apoptosis (98). Research showed that, after exposure to silica dust, all the levels of p53, plasminogen activator inhibitor-1, and apoptosis increased, and the levels of urokinase plasminogen activator decreased (99). TNF-α exerts an important influence on the pathogenesis of silicosis through NF-κB, which mediates the occurrence of apoptosis and inflammation (100). ER stress could attenuate the activation of caspase-12 and protein kinase RNA-like ER kinase (PERK)/eukaryotic initiation factor 2 α/C/EBP homologous protein pathways, thus inhibiting the silica-induced apoptosis (101).

### 7.4 Pyroptosis on the progression of pneumoconiosis

**Figure 4:**
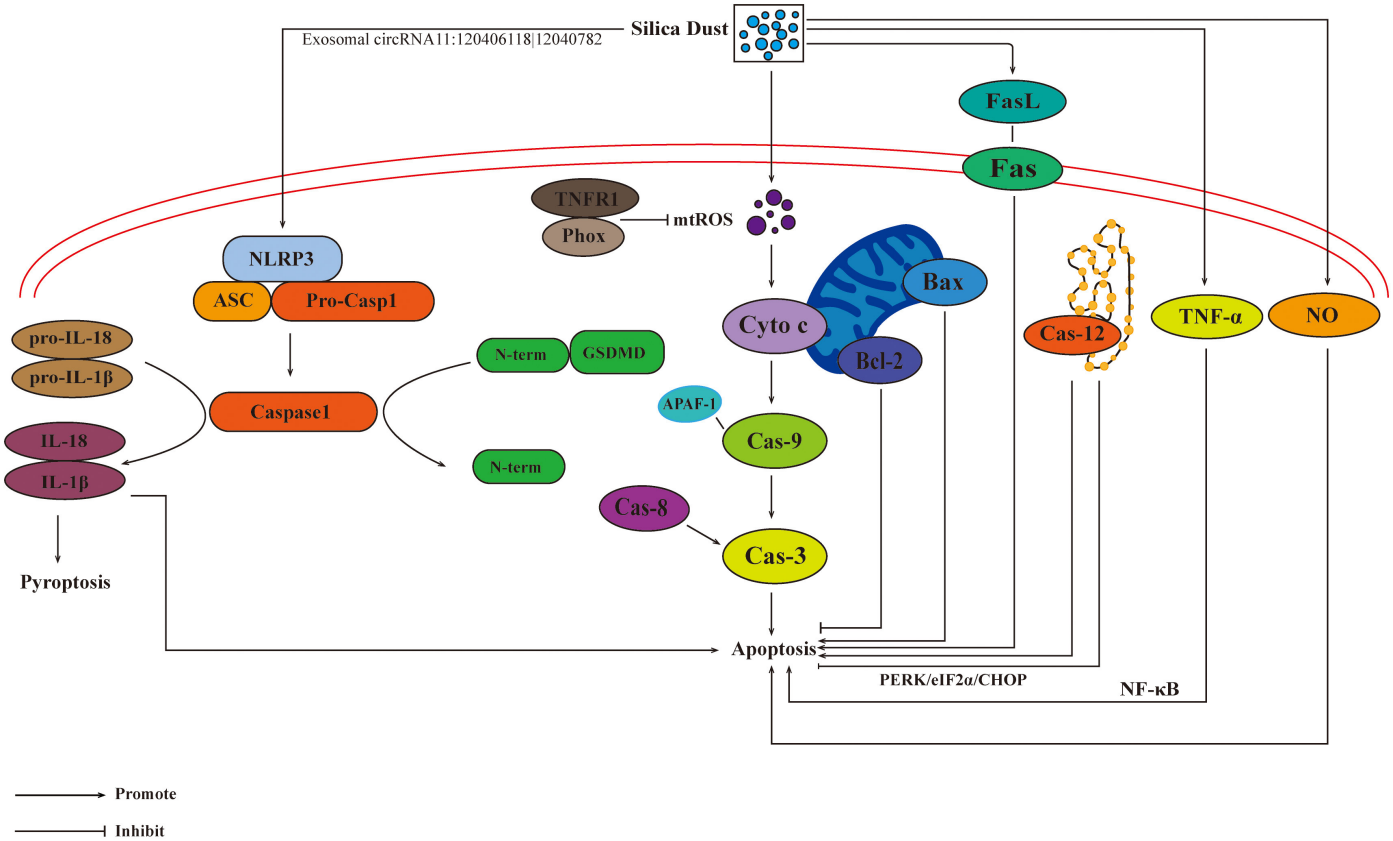
Influences of apoptosis and pyroptosis on the progression of pneumoconiosis. The activation of Fas/FasL pathway mediated by silica results in apoptosis. Cytochrome c can lead to the combination of APAF-1 and pro-caspase 9, resulting in the enzymatic cascade and apoptosis. Caspase-3 is activated by caspase-8, initiating a caspase-cascade and cell apoptosis. The inhibition of Caspase-12 results in apoptosis. TNFR1/Phox interaction inhibits the formation of mtROS, reducing macrophage apoptosis. TNF-α, NO, and exosomes are also involved in the development of apoptosis. Pyroptosis is mediated by the activation of NLRP3 inflammasome.

Pyroptosis is mediated by NOD-like receptor thermal protein domain associated protein 3 (NLRP3) inflammasome, a cytosolic multiprotein complex, which is composed of the innate immune receptor protein NLRP3, adapter protein apoptosis-associated speck-like protein containing a CARD, and inflammatory protease caspase-1. The assembled NLRP3 inflammasome can activate protease caspase-1, which promotes the release of IL-1β and IL-18 (102). Nalp3 inflammasome is associated with dust-induced pulmonary diseases, and it was reported to play a crucial role as a main proinflammatory “danger receptor” (103). Silica-induced activization of NLRP3 inflammasome was confirmed with co-localization of Caspase-1 and NLRP3, as well as increased levels of IL-1β and IL-18 (104). In addition, a new pathway of pyroptosis was discovered, and it was mediated by Caspase-3/-8/Gsdme pyroptotic pathways (105). Exosomal circRNA11:120406118|12040782 could facilitate NLRP3-induced macrophages pyroptosis (106), suggesting that more attention should be paid to the exosomes in terms of pathogenesis of pyroptosis. Under the influence of apotosis and pyroptosis, pathways and targets involved in the progression of pneumoconiosis are shown in Figure 4.

### 7.5 Epigenetics on the progression of pneumoconiosis

The role of epigenetics in the pathogenesis of silicosis should be studied extensively. Progression of silicosis may be caused by a combination of environmental and genetic factors. The relationship between genotype and phenotype, and the association between their correlation and disease susceptibility are very complicated due to the discrepancy in environment, lifestyle, and nutritional status (107). Recently, N-methyladenosine methylation has received considerable attention in the research on the progression of pneumoconiosis, and was found to be closely related to “phagosome,” “antigen processing and presentation„ and “apoptosis” (108). Environmental factors should be considered carefully, including smoking and bacteria.

TNF-α-308 G/A and−238A/G polymorphisms may be correlated with silicosis susceptibility, especially in Asians (109, 110). Telomerase gene variants and short telomeres may increase the susceptibility to silicosis, but do not affect the severity of the disease (111). The rs12812500 variant of the carboxypeptidase M gene may increase the susceptibility to silicosis (112). Genetic loss of Gas6 partly attenuates silica-induced autophagosomes accumulation (85), which may affect the progression of silicosis. Deficiency of RAB20 in macrophages/monocytes could promote the release of IL-1b and the activation of NLRP3 inflammasome, resulting in injury to the lysosome (113).

A recent study showed that smoking cessation could help reduce the risk of silicosis in silica-exposed workers (114). Nicotine, an addictive component in cigarettes, may induce apoptosis by blocking AM autophagic degradation of AM (115). LPS initiates the formation of autophagosomes through a Toll-like receptor 4 (TLR4)-dependent pathway and exacerbates apoptosis in AMs (116). LPS induces autophagy and apoptosis in macrophages. With the progression of silicosis, the level of Beclin 1 increased and the levels of the phosphorylation of MTOR, TLR4, MYD88, and TICAM1 decreased (116). A novel circRNA-SNP may increase the susceptibility to silicosis, so further investigations need to be conducted on the role of circRNAs in the progression of pneumoconiosis (117).

## 8 Therapeutic measures for pneumoconiosis

No cure is available for pneumoconiosis and most medical treatments can only decrease further lung damage and symptoms, underscoring the urgency of novel treatment modalities (2).

Whole lung lavage (WLL) can remove a certain amount of dust, cells, and soluble materials from the lungs, improving oxygen uptake and ventilatory efficiency in pneumoconiosis patients (118). However, negative suction pressure will cause different degrees of lung damage during WLL. Additionally, pulmonary function parameters were reported to worsen after WLL, including forced expiratory volume, residual volume, and diffusing capacity of the lungs for carbon monoxide (119).

Since there are no effective treatments available for end-stage pneumoconiosis, lung transplant is the only option for patients with fatal respiratory failure (5). However, lung transplantation has some limitations, including donor shortage, proper selection of candidates, primary graft dysfunction, and chronic lung allograft dysfunction (120). Meanwhile, lung transplant recipients were reported to have a short median survival time of only 6–7 years (6).

### 8.1 Promising targets for pneumoconiosis

An increasing amount of evidence has demonstrated that the dysregulation of miRNAs may play an important role in the progression of pneumoconiosis (121). miRNAs play an important role in the progression of pneumoconiosis, and they have emerged as potent regulators of EMT and mesenchymal epithelial transition (MET). A recent study proved that miR-770-5p suppressed the activation of pulmonary fibroblasts and further inhibited silica-induced pulmonary fibrosis by targeting transforming growth factor beta receptors (TGFBR1). It was reported that transduction of TGF-β1 signaling pathway decreased TGFBR1, and the activation of MRC-5 cells was inhibited after TGFBR1 was knocked out, while increase in the growth of these cells was observed after the overexpression of TGFBR1 (121). Spouty1 (SPRY1) is identified as the target gene of miR-7219-3p, and its knockout or overexpression could promote or inhibit fecal microbiota transplantation (FMT), respectively, via the Ras/ERK/MAPK signaling pathway. Therefore, miR-7219-3p could be deemed a novel therapeutic target for pneumoconiosis treatment (122).

ZC3H4 participates in macrophage activation and EMT. Research has confirmed that ZC3H4 participates in the silica-induced EndoMT via ER stress and autophagy, indicating the possibility of treating pneumoconiosis by targeting ZC3H4 (89). According to a new study, A2a receptor (A2aR) could reverse EMT by mediating Wnt/β-catenin pathway and inhibit the development of silicosis (123). Glycolytic reprogramming is an important metabolic feature of the progression of pulmonary fibrosis (124), however, the specific mechanism of glycolysis in silicosis is still unclear. A study have confirmed that N-acetyl-seryl-aspartyl-lysyl-proline (Ac-SDKP) treatment can inhibit glycolytic reprogramming in silica-induced lung macrophages and alleviate pulmonary fibrosis (125).

CD44-RhoA-YAP signaling is involved in mechanics-induced fibroblast activation, therefore, fibrosis in pneumoconiosis could be reversed by targeting this signaling pathway (78). Activating associated autophagy pathways, such as PI3K/Akt/mTOR and Gas6/Mer-mediated autophagy signaling pathway, is proved to have therapeutic effect on pneumoconiosis (83, 85). Fas/FasL pathway may be involved in the progression of AM apoptosis, suggesting that silicosis could be prevented or treated by inhibiting this signaling pathway (46).

Lymphatic vessels are beneficial to the removal of silica dust and the suppression of inflammation (126). Promoting the formation of lymphatic vessels is helpful in the early prevention and treatment of pneumoconiosis (126). Ginsenoside Rg1 promoted lymphatic transport in silicotic rats through vascular endothelial growth factor C/vascular endothelial growth factor receptor 3 signaling pathway, exerting a protective influence on lung burden of silica (127). Specific therapeutic targets for pneumoconiosis treatment are listed in Table 4.

**Table 4:** New therapeutic targets for pneumoconiosis.

**Targets**	**Expression level**	**Mechanisms**	**Effects**	**References**
miR-449a	Up	Promotes autophagy	Ameliorates pulmonary fibrosis	(128)
miR-205-5p	Up	Promotes autophagy by inhibiting S-phase kinase-associated protein 2-mediated Beclin1	Ameliorates pulmonary fibrosis	(129)
miR-29b	Up	Promotes MET and by suppressing EMT	Ameliorates pulmonary fibrosis ang pulmonary function	(130)
miR-770-5p	Up	Targets TGFBR1	Ameliorates pulmonary fibrosis	(121)
miR-411-3p	Up	Inhibits MRTF-A/SRF signaling	Ameliorates pulmonary fibrosis	(131)
SPRY1	Down	Inhibits the Ras/ERK/MAPK signaling pathway and reduces the expression of miR-7219-3p	Inhibits FMT and pulmonary fibrosis	(122)
miR-326	Up	Promotes autophagy by TNF superfamily14 and polypyrimidine tract-binding protein 1	Ameliorates pulmonary fibrosis	(132)
ZC3H4	Down	Regulates ER stress and autophagy	Ameliorates pulmonary fibrosis	(89, 90)
RhoGDIα	Down	Inhibits myofibroblast trans-differentiation	Ameliorates pulmonary fibrosis	(133)
IgE and FcεRI	Down	Reduce the role IgE and FcεRI play	Ameliorates pulmonary fibrosis	(134)
BCL2 binding component 3	Down	Inhibits autophagy	Ameliorates pulmonary fibrosis	(135)
Cav-1	Up	Inhibited infiltration of inflammatory cells and secretion of inflammatory factors	Ameliorates pulmonary inflammation and fibrosis	(136)
A2aR	Up	Reverses the EMT process	Ameliorates pulmonary fibrosis	(123)
CD44-RhoA-YAP signaling	Inhibit	Inhibits fibroblast activation	Ameliorates pulmonary fibrosis	(78)

### 8.2 Promising drugs for pneumoconiosis

Drugs used to treat other diseases, such as corticosteroids and amiodarone, could also be used in the management of pneumoconiosis (137, 138). Amiodarone, a unique antiarrhythmic agent, is effective in the treatment of a wide range of rhythm abnormalities (139). With the ability to inhibit the activity of AM and the whole lung phospholipase, amiodarone can increase the amount of phospholipids in lung cells, airways, and alveoli (138). A previous study showed that the increase in pulmonary phospholipid reduced the acute damage caused by intratracheal instillation of silica in rats, indicating that amiodarone can attenuate acute damage in lungs by increasing the content of phospholipids (138).

In addition, some drugs extracted from natural plants with anti-inflammatory and anti-fiber properties can also be used to treat pneumoconiosis, including anti-snake venom injection (140), dihydrotanshinone I (141), and intranasal curcumin (142). Dioscin, a steroidal saponin, reduces the recruitment of fibrocytes and inhibits TNFβ/Smad3 signaling, which greatly suppresses the activation of fibroblasts (143), and it could alleviate pulmonary inflammation and fibrosis by promoting autophagy and reducing apoptosis of AMs (144). Dihydroquercetin, a flavonoid compound with anti-inflammatory property, could be used for pneumoconiosis treatment because it inhibits ferritinophagy-mediated human bronchial epithelial cells ferroptosis and alleviates pulmonary fibrosis (145). Specific drugs are listed in Table 5.

**Table 5:** Prospective drugs for pneumoconiosis.

**Drugs**	**Mechanisms**	**Involved signaling pathways**	**Effects**	**References**
Pirfenidone	Inhibits the secretion of IL-17A Inhibits macrophage polarization Inhibits epithelial-mesenchymal transition	TGF-β1/Smad pathway JAK2/STAT3 signaling pathway	Ameliorates pulmonary inflammation and fibrosis	(146–148)
Fostamatinib	Targets SYK	Not reported	Ameliorates pulmonary inflammation and fibrosis	(149)
Gefitinib	Targets EGFR	Not reported	Ameliorates pulmonary inflammation and fibrosis	(149)
Metformin	Activates autophagy	AMPK-mTOR signaling pathway	Attenuates pulmonary fibrosis	(84)
Ramatroban	Blocks the receptors of PGD and TXA	Not reported	Inhibits the progression of silicosis	(41)
Astragaloside IV	Reduces the expression of Collagen I, fibronectin and α-SMA	TGF-β1/Smad signaling pathway	Attenuates pulmonary fibrosis	(140)
Intranasal curcumin	Reduces airway inflammation and structural changes	Not reported	Ameliorates lung damage	(142)
Dihydrotanshinone I	Regulates the Th immune response Inhibits STAT1 and STAT3	Not reported	Ameliorates pulmonary inflammation	(141)
Carvedilol	Underlies inflammatory and fibrotic sequel	P-AKT/mTOR/TGFβ1 signaling	Ameliorates pulmonary fibrosis	(150)
Tetrandrine	Inhibits both the canonical and non-canonical NLRP3 inflammasome pathways	Canonical and non-canonical NLRP3 inflammasome pathways	Ameliorates pulmonary inflammation and fibrosis	(151)
Dioscin	Modulates innate and adaptive immune responses Reduces the recruitment of fibrocytes, protected epithelial cells Inhibits fibroblast activation Promotes autophagy Reduces apoptosis	TNFβ/Smad3 signaling	Ameliorates pulmonary inflammation and fibrosis	(143, 144)
Atractylenolide III	Inhibits autophagy	mTOR-dependent signaling pathway	Alleviate the apoptosis of AMs	(152)
Tanshinone IIA	Decreases the expression of collagen I, fibronectin and α-SMA Reduces NADPH oxidase 4 expression	TGF-β1/Smad signaling Nrf2/ARE pathway	Ameliorates pulmonary inflammation, structural damage and fibrosis	(153)
Glycyrrhizic acid	Inhibits the interaction between HMGB1 and BRG1	PI3K/Akt/mTOR pathway	Ameliorates pulmonary fibrosis	(154)

### 8.3 Other therapies for pneumoconiosis

Systemic administration of Mesenchymal stem cells (MSCs) was reported to ameliorate lung inflammation and attenuate fibrosis in experimental silicosis, and could be used as an emerging treatment for pulmonary fibrosis (155). The group with adipose-derived MSC (AD-MSCs) transplantation showed a significant increase in Bcl-2/Bax ratio and a drastic reduction in the inflammatory response and Caspase-3 protein expression, implying that AD-MSCs may slow the development of silicosis by influencing inflammation and apoptosis (156). Bone marrow mononuclear cells significantly alleviate pulmonary inflammation and fibrosis, especially when transplanted from healthy individuals (157). However, the direct use of stem cells for therapeutic purposes has remained limited due to several factors, such as safety and high expenditure. Magnetic targeting (MT) is regarded as a potential means of prolonging MSC retention in the lungs to improve their beneficial effects, indicating that MT could be adopted as a prospective strategy to enhance MSC therapies for pneumoconiosis (155).

Exosomes secreted from stem cells hold great therapeutic potential with the added advantage of being free from the restrictions of cell-based therapy. Exosomes derived from human umbilical cord mesenchymal stem cells (hucMSC-Exos) were reported to play a potential role in improving pulmonary fibrosis (158), since they transfer let-7i-5p to inhibit the activation of fibroblasts and then alleviate pulmonary fibrosis through the TGFBR1/Smad3 signaling pathway (159). MSC-derived extracellular vesicles (MSC-derived EVs) also have similar therapeutic potential in treating pneumoconiosis (160).

Hepatocyte growth factor (HGF) is a potential anti-inflammatory and anti-fibrotic growth factor (161), and it was reported to play a significant role in pulmonary tissue repair in adults (162). Administration of HGF protein or ectopic expression of HGF induced normal tissue repair and prevented fibrotic remodeling in animal models of pulmonary fibrosis (162). HGF inhibits fibrotic remodeling, which is mediated by multiple direct and indirect mechanisms, including the induction of cell survival, proliferation of pulmonary epithelial and endothelial cells, and reduction of myofibroblast accumulation (162), suggesting that HGF can be used to treat pneumoconiosis. HGF could also be applied to polyethyleneimine-polyethylene glycol/plasmid encoding human HGF gene/hyaluronic acid (PEG-PEI/pHGF/HA) nanoparticles carrying HGF gene through chemical synthesis; pHGF was delivered into the lungs of silicostic mice effectively via PEG-PEI(HA), and it resulted in a decrease in inflammation and collagen deposition in the lungs. Therefore, gene therapy with PEG-PEI/pHGF/HA nanoparticles is a promising strategy for the treatment of silicosis, and it would provide research foundation and novel ideas for the treatment of silicosis (163). The combination of MSC and HGF has been reported to have good therapeutic effect on some pneumoconiosis patients (164).

Nanotechnology can be integrated with existing drugs for the treatment of pneumoconiosis or be applied to the development of new therapies, which will make drugs more effective and reduce the undesirable side effects, and the therapeutic goals can be better achieved. Compared with stand-alone drugs in traditional respiratory therapy, the integration of drugs and different nanostructures showed a better drug bioavailability, transport, and delivery (165). A study reported that fullerene nanoparticles (FNs) could effectively inhibit the activation of NLRP3 inflammasome, which could prevent the secretion of mature IL-1β and neutrophil influx due to its superior ROS scavenging capability. Importantly, FNs did not cause any obvious toxicity after pulmonary administration (166). New nanotechnologies such as FNs will be a boon to the treatment of pneumoconiosis in the future.

## 9 Prospects of pneumoconiosis

Further research needs to be conducted on the pathogenesis of pneumoconiosis to find novel and accurate targets for diagnosis and to develop new therapeutic approaches. Recently, increasing research has revealed that autophagy, apoptosis and pyroptosis are involved in the development of pneumoconiosis to a certain extent (166). The occurrence of pneumoconiosis is dependent on the interaction between three above-mentioned phenomena (167), and molecules and signaling pathways involved in these processes can be used as diagnostic and therapeutic targets for pneumoconiosis. Additionally, the mechanism of pneumoconiosis should be considered from the perspective of the interplay between genetic and environmental factors (107). More environmental factors and relevant genes are expected to be discovered in future studies, which would help to explain the pathogenesis of pneumoconiosis more clearly. As carriers, exosomes could transport specific cargoes such as nucleic acids, lipids and proteins, and promote biological process and idiopathic pulmonary fibrosis (82). Further research on the role of exosomes in pathogenesis of pneumoconiosis would uncover novel promising biomarkers, therapeutic targets, and relevant drugs.

Developing effective specific models such as pre-clinical and human organoid-based models is significant for further exploration on the mechanisms of pneumoconiosis (36). Like any other controllable model organisms, complex patient-derived mannequin systems will consequently become powerful research tools for understanding human physiology and disease development (168). Besides, it is equally important to apply various new research techniques to the field of pneumoconiosis because a single technique alone fails to explain the mechanism of this disease in most of the cases. For example, integrative omics plays a key role in the prediction and early diagnosis of pneumoconiosis as well as in the treatment and prognosis (169). Multi-omics approach was used and it was found that mA methylation played an essential role in the occurrence of silicosis. Multi-omics approach could be used as a novel and viable strategy for the prevention and treatment of silicosis. These approaches have paved the way for clarifying the epigenetic mechanisms underlying the pathogenesis of silicosis (108). Rapid development of integrative omics such as genomics, transcriptomics, proteomics, and metabolomics has revealed the differences among individuals indicating that personalized medicine has great application prospect. Personalized medicine would provide specific individuals with interventions for their diseases, and the treatments can be tailored to their nuanced needs caused by the differences in the levels of molecule, physiology, environmental exposure, and behavior.

New lung scanning methods, including HRCT, EIT, and magneto pneumography, have improved the reliability of diagnosis of pneumoconiosis, making early diagnosis of pneumoconiosis possible (58, 59, 62). Standardized techniques, coordination, and consensus should be adopted to promote the clinical application of these imaging methods (60). AI, a hot topic in medical imaging, is a promising method in the diagnosis of pneumoconiosis, for it can develop diagnostic algorithms in an innovative way with the advantage of evaluating multiple issues rapidly (63). In addition, it is important to find novel biomarkers for early and accurate diagnosis of pneumoconiosis (2). Existing methods, such as WLL, cause damage to human body, though they can alleviate the progression of pneumoconiosis (119). Several drugs with anti-inflammatory and anti-fiber properties have been found useful in the treatment of pneumoconiosis. Researchers are expected to develop more new drugs that can treat pneumoconiosis effectively (88). Being adult stem cells, MSCs have shown promising results in the treatment of pneumoconiosis (156), with strong differentiation ability and immune regulation function (170). Importantly, the secretory factors produced by MSCs play critical roles in tissue repair, which support both engraftment and trophic functions (autocrine and paracrine) (170). With these features, MSCs have been increasingly utilized in clinical trials for cell replacement and immune suppression, and they are considered promising in the treatment of pneumoconiosis. It is worth noting that HGF's anti-inflammatory and anti-fibrotic attributes make it possible to treat pneumoconiosis and alleviate its progression (162). The combination of HGF with nanotechnology or MSCs will be of much significance to the treatment of pneumoconiosis (163, 164).

## References

[1] Bell JL Mazurek JM. Trends in pneumoconiosis deaths - United States, 1999-2018. MMWR Morb Mortal Wkly Rep. (2020) 69:693–8. 10.15585/mmwr.mm6923a1 32525855 PMC7315788

[2] Li J Yin P Wang H Wang L You J Liu J. The burden of pneumoconiosis in China: an analysis from the global burden of disease study 2019. BMC Public Health. (2022) 22:1114. 10.1186/s12889-022-13541-x 35659279 PMC9166455

[3] Laney AS Petsonk EL Wolfe AL Attfield MD. Comparison of storage phosphor computed radiography with conventional film-screen radiography in the recognition of pneumoconiosis. Eur Respir J. (2010) 36:122–7. 10.1183/09031936.00127609 19926739

[4] De Vuyst P Camus P. The past and present of pneumoconioses. Curr Opin Pulm Med. (2000) 6:151–6. 10.1097/00063198-200003000-00012 10741776

[5] Barnes H Goh NSL Leong TL Hoy R. Silica-associated lung disease: an old-world exposure in modern industries. Respirology. (2019) 24:1165–75. 10.1111/resp.13695 31517432

[6] Hoy RF Chambers DC. Silica-related diseases in the modern world. Allergy. (2020) 75:2805–17. 10.1111/all.14202 31989662

[7] Ding Q Schenk L Hansson SO. Occupational diseases in the People's Republic of China between 2000 and 2010. Am J Ind Med. (2013) 56:1423–32. 10.1002/ajim.22245 23970481

[8] Leung CC Yu IT Chen W. Silicosis. Lancet. (2012) 379:2008–18. 10.1016/S0140-6736(12)60235-9 22534002

[9] Yamano S Goto Y Takeda T Hirai S Furukawa Y Kikuchi Y. Pulmonary dust foci as rat pneumoconiosis lesion induced by titanium dioxide nanoparticles in 13-week inhalation study. Part Fibre Toxicol. (2022) 19:58. 10.1186/s12989-022-00498-3 36100920 PMC9472424

[10] Rivera-Ortega P Molina-Molina M. Interstitial lung diseases in developing countries. Ann Glob Health. (2019) 85:4. 10.5334/aogh.2414 30741505 PMC7052338

[11] Chair SY Chan JYW Law BMH Waye MMY Chien WT. Genetic susceptibility in pneumoconiosis in China: a systematic review. Int Arch Occup Environ Health. (2023) 96:45–56. 10.1007/s00420-022-01893-1 35906431

[12] Diseases GBD Injuries C. Global burden of 369 diseases and injuries in 204 countries and territories, 1990-2019: a systematic analysis for the global burden of disease study 2019. Lancet. (2020) 396:1204–22. 10.1016/S0140-6736(20)30925-9 33069326 PMC7567026

[13] Shi P Xing X Xi S Jing H Yuan J Fu Z. Trends in global, regional and national incidence of pneumoconiosis caused by different aetiologies: an analysis from the global burden of disease study 2017. Occup Environ Med. (2020) 77:407–14. 10.1136/oemed-2019-106321 32188634

[14] Yang M Wang D Gan S Fan L Cheng M Yu L. Increasing incidence of asbestosis worldwide, 1990-2017: results from the global burden of disease study 2017. Thorax. (2020) 75:798–800. 10.1136/thoraxjnl-2020-214822 32467338

[15] Blackley DJ Halldin CN Laney AS. Continued increase in prevalence of coal workers' pneumoconiosis in the United States, 1970-2017. Am J Public Health. (2018) 108:1220–2. 10.2105/AJPH.2018.304517 30024799 PMC6085042

[16] Liu T Liu S. The impacts of coal dust on miners' health: a review. Environ Res. (2020) 190:109849. 10.1016/j.envres.2020.109849 32763275

[17] Wu N Xue C Yu S Ye Q. Artificial stone-associated silicosis in China: a prospective comparison with natural stone-associated silicosis. Respirology. (2020) 25:518–24. 10.1111/resp.13744 31828940 PMC7187561

[18] The L. Improving occupational health in China. Lancet. (2019) 394:443. 10.1016/S0140-6736(19)31799-4 31402011

[19] Zhang Z Zhao Y Sun D. China's occupational health challenges. Occup Med. (2017) 67:87–90. 10.1093/occmed/kqw102 28393171

[20] Voelker R. Black lung resurgence raises new challenges for coal country physicians. JAMA. (2019) 321:17–9. 10.1001/jama.2018.15966 30540357

[21] Barmania S. Deadly denim: sandblasting-induced silicosis in the jeans industry. Lancet Respir Med. (2016) 4:543. 10.1016/S2213-2600(16)30102-3 27155769

[22] Wan X Zhang X Pan W Liu B Yu L Wang H. Ratiometric fluorescent quantification of the size-dependent cellular toxicity of silica nanoparticles. Anal Chem. (2019) 91:6088–96. 10.1021/acs.analchem.9b00633 31001976

[23] Zou H Shi Z Zhang Y Zhou J Fang X Zhang Y. Epidemiological characteristics and survival analysis on patients with occupational pneumoconiosis in Zhejiang Province from 1987 to 2019. Front Public Health. (2022) 10:1006391. 10.3389/fpubh.2022.1006391 36311604 PMC9614357

[24] Cohen RA Petsonk EL Rose C Young B Regier M Najmuddin A. Lung pathology in US coal workers with rapidly progressive pneumoconiosis implicates silica and silicates. Am J Respir Crit Care Med. (2016) 193:673–80. 10.1164/rccm.201505-1014OC 26513613 PMC4824937

[25] Bazaluk O Cheberiachko S Cheberiachko Y Deryugin O Lozynskyi V Knysh I. Development of a dust respirator by improving the half mask frame design. Int J Environ Res Public Health. (2021) 18:5482. 10.3390/ijerph18105482 34065447 PMC8160864

[26] Liao X Wang B Wang L Zhu J Chu P Zhu Z. Experimental study on the wettability of coal with different metamorphism treated by surfactants for coal dust control. ACS Omega. (2021) 6:21925–38. 10.1021/acsomega.1c02205 34497888 PMC8412940

[27] Wang P Shen S Zhou L Liu D. Turbulent aggregation and deposition mechanism of respirable dust pollutants under wet dedusting using a two-fluid model with the population balance method. Int J Environ Res Public Health. (2019) 16:3359. 10.3390/ijerph16183359 31514472 PMC6765917

[28] Qian J Wang J Liu H Xu H. Numerical investigation of fine particulate matter aggregation and removal by water spray using swirling gas flow. Int J Environ Res Public Health. (2022) 19:16129. 10.3390/ijerph192316129 36498216 PMC9740401

[29] Jing D Liu H Zhang T Ge S Ren S Ma M. Study on coal dust diffusion law and new pneumatic spiral spray dedusting technology at transfer point of mine cross roadway. PLoS ONE. (2022) 17:e0272304. 10.1371/journal.pone.0272304 35994466 PMC9394847

[30] Peng H Nie W Cai P Liu Q Liu Z Yang S. Development of a novel wind-assisted centralized spraying dedusting device for dust suppression in a fully mechanized mining face. Environ Sci Pollut Res Int. (2019) 26:3292–307. 10.1007/s11356-018-3264-8 30267349

[31] Zeng F Jiang Z Wang Y. Study on the control of high ore pass dust pollution by pre-injection foam dedusting technology in the ore bin. Environ Sci Pollut Res Int. (2023) 30:606–21. 10.1007/s11356-022-22164-z 35904737

[32] Rose C Heinzerling A Patel K Sack C Wolff J Zell-Baran L. Severe silicosis in engineered stone fabrication workers - California, Colorado, Texas, and Washington, 2017-2019. MMWR Morb Mortal Wkly Rep. (2019) 68:813–8. 10.15585/mmwr.mm6838a1 31557149 PMC6762184

[33] Krabbe J Steffens KM Drießen S Kraus T. Lung cancer risk and occupational pulmonary fibrosis: systematic review and meta-analysis. Eur Respir Rev. (2024) 33:230224. 10.1183/16000617.0224-2023 38355151 PMC10865097

[34] Pollard KM. Silica, silicosis, and autoimmunity. Front Immunol. (2016) 7:97. 10.3389/fimmu.2016.00097 27014276 PMC4786551

[35] Cowie RL Hay M Thomas RG. Association of silicosis, lung dysfunction, and emphysema in gold miners. Thorax. (1993) 48:746–9. 10.1136/thx.48.7.746 8153925 PMC464663

[36] Perret JL Plush B Lachapelle P Hinks TS Walter C Clarke P. Coal mine dust lung disease in the modern era. Respirology. (2017) 22:662–70. 10.1111/resp.13034 28370783

[37] Qi XM Luo Y Song MY Liu Y Shu T Liu Y. Pneumoconiosis: current status and future prospects. Chin Med J (Engl). (2021) 134:898–907. 10.1097/CM9.0000000000001461 33879753 PMC8078400

[38] Lee JS Shin JH Lee JO Lee KM Kim JH Choi BS. Levels of exhaled breath condensate ph and fractional exhaled nitric oxide in retired coal miners. Toxicol Res. (2010) 26:329–37. 10.5487/TR.2010.26.4.329 24278541 PMC3834506

[39] Chong S Lee KS Chung MJ Han J Kwon OJ Kim TS. Pneumoconiosis: comparison of imaging and pathologic findings. Radiographics. (2006) 26:59–77. 10.1148/rg.261055070 16418244

[40] Sato T Takeno M Honma K Yamauchi H Saito Y Sasaki T. Heme oxygenase-1, a potential biomarker of chronic silicosis, attenuates silica-induced lung injury. Am J Respir Crit Care Med. (2006) 174:906–14. 10.1164/rccm.200508-1237OC 16858012

[41] Pang J Qi X Luo Y Li X Shu T Li B. Multi-omics study of silicosis reveals the potential therapeutic targets Pgd(2) and Txa(2). Theranostics. (2021) 11:2381–94. 10.7150/thno.47627 33500731 PMC7797695

[42] Kim KA Lim Y Kim JH Kim EK Chang HS Park YM. Potential biomarker of coal workers' pneumoconiosis. Toxicol Lett. (1999) 108:297–302. 10.1016/S0378-4274(99)00101-0 10511274

[43] Chan JYW Tsui JCC Law PTW So WKW Leung DYP Sham MMK. Rna-Seq revealed Atf3-regulated inflammation induced by silica. Toxicology. (2018) 393:34–41. 10.1016/j.tox.2017.11.001 29102675

[44] Zhou Z Jiang R Yang X Guo H Fang S Zhang Y. Circrna mediates silica-induced macrophage activation via Hectd1/Zc3h12a-dependent ubiquitination. Theranostics. (2018) 8:575–92. 10.7150/thno.21648 29290828 PMC5743568

[45] Lee S Honda M Yamamoto S Kumagai-Takei N Yoshitome K Nishimura Y. Role of nephronectin in pathophysiology of silicosis. Int J Mol Sci. (2019) 20:2581. 10.3390/ijms20102581 31130697 PMC6566895

[46] Yao SQ Rojanasakul LW Chen ZY Xu YJ Bai YP Chen G. Fas/Fasl pathway-mediated alveolar macrophage apoptosis involved in human silicosis. Apoptosis. (2011) 16:1195–204. 10.1007/s10495-011-0647-4 21910009 PMC4707682

[47] Cui J Guan Q Lv H Fu K Fu R Feng Z. Three-dimensional nanorod array for label-free surface-enhanced raman spectroscopy analysis of microrna pneumoconiosis biomarkers. Spectrochim Acta A Mol Biomol Spectrosc. (2021) 261:120015. 10.1016/j.saa.2021.120015 34098483

[48] Huang R Yu T Li Y Hu J. Upregulated Has-Mir-4516 as a potential biomarker for early diagnosis of dust-induced pulmonary fibrosis in patients with pneumoconiosis. Toxicol Res (Camb). (2018) 7:415–22. 10.1039/C8TX00031J 30090591 PMC6060724

[49] Xia J Wang D Guo W Pei Y Zhang L Bao L. Exposure to micron-grade silica particles triggers pulmonary fibrosis through cell-to-cell delivery of exosomal Mir-107. Int J Biol Macrom. (2024) 266:131058. 10.1016/j.ijbiomac.2024.131058 38522707

[50] Chorley BN Atabakhsh E Doran G Gautier JC Ellinger-Ziegelbauer H Jackson D. Methodological considerations for measuring biofluid-based microrna biomarkers. Crit Rev Toxicol. (2021) 51:264–82. 10.1080/10408444.2021.1907530 34038674 PMC8577439

[51] Amrovani M Mohammadtaghizadeh M Aghaali MK Zamanifard S Alqasi A Sanei M. Long non-coding rnas: potential players in cardiotoxicity induced by chemotherapy drugs. Cardiovasc Toxicol. (2022) 22:191–206. 10.1007/s12012-021-09681-y 34417760

[52] Cai W Xu H Zhang B Gao X Li S Wei Z. Differential expression of lncrnas during silicosis and the role of Loc103691771 in myofibroblast differentiation induced by Tgf-Beta1. Biomed Pharmacother. (2020) 125:109980. 10.1016/j.biopha.2020.109980 32028236

[53] Ma J Zhou Y Li W Xiao L Yang M Tan Q. Association between plasma hmgb-1 and silicosis: a case-control study. Int J Mol Sci. (2018) 19:4043. 10.3390/ijms19124043 30558126 PMC6320808

[54] Benmerzoug S Rose S Bounab B Gosset D Duneau L Chenuet P. Sting-dependent sensing of Self-DNA drives silica-induced lung inflammation. Nat Commun. (2018) 9:5226. 10.1038/s41467-018-07425-1 30523277 PMC6283886

[55] Xue C Wu N Li X Qiu M Du X Ye Q. Serum concentrations of krebs von den lungen-6, surfactant protein d, and matrix metalloproteinase-2 as diagnostic biomarkers in patients with asbestosis and silicosis: a case-control study. BMC Pulm Med. (2017) 17:144. 10.1186/s12890-017-0489-0 29149883 PMC5693552

[56] Chen Z Shi J Zhang Y Zhang J Li S Guan L. Screening of serum biomarkers of coal workers' pneumoconiosis by metabolomics combined with machine learning strategy. Int J Environ Res Public Health. (2022) 19:7051. 10.3390/ijerph19127051 35742299 PMC9222502

[57] Cheng Z Zhang Y Wu S Zhao R Yu Y Zhou Y. Peripheral blood circular Rna Hsa_Circ_0058493 as a potential novel biomarker for silicosis and idiopathic pulmonary fibrosis. Ecotoxicol Environ Saf. (2022) 236:113451. 10.1016/j.ecoenv.2022.113451 35378401

[58] Talini D Paggiaro PL Falaschi F Battolla L Carrara M Petrozzino M. Chest radiography and high resolution computed tomography in the evaluation of workers exposed to silica dust: relation with functional findings. Occup Environ Med. (1995) 52:262–7. 10.1136/oem.52.4.262 7795742 PMC1128205

[59] Ke XY Hou W Huang Q Hou X Bao XY Kong WX. Advances in electrical impedance tomography-based brain imaging. Mil Med Res. (2022) 9:10. 10.1186/s40779-022-00370-7 35227324 PMC8883715

[60] Frerichs I Amato MB van Kaam AH Tingay DG Zhao Z Grychtol B. Chest electrical impedance tomography examination, data analysis, terminology, clinical use and recommendations: consensus statement of the translational eit development study group. Thorax. (2017) 72:83–93. 10.1136/thoraxjnl-2016-208357 27596161 PMC5329047

[61] Kircher M Elke G Stender B Hernandez Mesa M Schuderer F Dossel O. Regional lung perfusion analysis in experimental ards by electrical impedance and computed tomography. IEEE Trans Med Imaging. (2021) 40:251–61. 10.1109/TMI.2020.3025080 32956046

[62] Le Gros V Lemaigre D Suon C Pozzi JP Liot F. Magnetopneumography: a general review. Eur Respir J. (1989) 2:149–59. 10.1183/09031936.93.02020149 2649394

[63] Kaplan A Cao H FitzGerald JM Iannotti N Yang E Kocks JWH. Artificial intelligence/machine learning in respiratory medicine and potential role in asthma and copd diagnosis. J Allergy Clin Immunol Pract. (2021) 9:2255–61. 10.1016/j.jaip.2021.02.014 33618053

[64] Chassagnon G Vakalopoulou M Paragios N Revel M. Artificial intelligence applications for thoracic imaging. Eur J Radiol. (2020) 123:108774. 10.1016/j.ejrad.2019.108774 31841881

[65] Hao C Jin N Qiu C Ba K Wang X Zhang H. Balanced convolutional neural networks for pneumoconiosis detection. Int J Environ Res Public Health. (2021) 18:9091. 10.3390/ijerph18179091 34501684 PMC8431598

[66] Zhang L Rong R Li Q Yang DM Yao B Luo D. A deep learning-based model for screening and staging pneumoconiosis. Sci Rep. (2021) 11:2201. 10.1038/s41598-020-77924-z 33500426 PMC7838184

[67] Koul A Bawa RK Kumar Y. Artificial intelligence techniques to predict the airway disorders illness: a systematic review. Arch Comput Methods Eng. (2022) 30:831–64. 10.1007/s11831-022-09818-4 36189431 PMC9516534

[68] Thakur SA Beamer CA Migliaccio CT Holian A. Critical role of marco in crystalline silica-induced pulmonary inflammation. Toxicol Sci. (2009) 108:462–71. 10.1093/toxsci/kfp011 19151164 PMC2664690

[69] Biswas R Hamilton RF Jr Holian A. Role of lysosomes in silica-induced inflammasome activation and inflammation in absence of marco *J Immunol Res*. (2014) 2014:304180. 10.1155/2014/304180 25054161 PMC4099041

[70] Fubini B Hubbard A. Reactive oxygen species (ROS) and reactive nitrogen species (RNS) generation by silica in inflammation and fibrosis. Free Radic Biol Med. (2003) 34:1507–16. 10.1016/S0891-5849(03)00149-7 12788471

[71] Zhao Y Hao C Bao L Wang D Li Y Qu Y. Silica particles disorganize the polarization of pulmonary macrophages in mice. Ecotoxicol Environ Saf. (2020) 193:110364. 10.1016/j.ecoenv.2020.110364 32114243

[72] Fu R Li Q Fan R Zhou Q Jin X Cao J. Itraq-based secretome reveals that sio(2) induces the polarization of raw2647 macrophages by activation of the Nod-Rip2-Nf-Kappab signaling pathway. Environ Toxicol Pharmacol. (2018) 63:92–102. 10.1016/j.etap.2018.08.010 30189374

[73] Liu TT Sun HF Han YX Zhan Y Jiang JD. The role of inflammation in silicosis. Front Pharmacol. (2024) 15:1362509. 10.3389/fphar.2024.1362509 38515835 PMC10955140

[74] Srivastava KD Rom WN Jagirdar J Yie TA Gordon T Tchou-Wong KM. Crucial role of interleukin-1beta and nitric oxide synthase in silica-induced inflammation and apoptosis in mice. Am J Respir Crit Care Med. (2002) 165:527–33. 10.1164/ajrccm.165.4.2106009 11850347

[75] Kumar A Abdelmalak B Inoue Y Culver DA. Pulmonary alveolar proteinosis in adults: pathophysiology and clinical approach. Lancet Respir Med. (2018) 6:554–65. 10.1016/S2213-2600(18)30043-2 29397349

[76] Jouneau S Menard C Lederlin M. Pulmonary alveolar proteinosis. Respirology. (2020) 25:816–26. 10.1111/resp.13831 32363736

[77] Li J Yao W Hou JY Zhang L Bao L Chen HT. The role of fibrocyte in the pathogenesis of silicosis. Biomed Environ Sci. (2018) 31:311–6. 10.3967/bes2018.040 29773095

[78] Li S Li C Zhang Y He X Chen X Zeng X. Targeting mechanics-induced fibroblast activation through CD44-Rhoa-Yap pathway ameliorates crystalline silica-induced silicosis. Theranostics. (2019) 9:4993–5008. 10.7150/thno.35665 31410197 PMC6691376

[79] Xu Q Cheng D Li G Liu Y Li P Sun W. Circhipk3 regulates pulmonary fibrosis by facilitating glycolysis in Mir-30a-3p/Foxk2-dependent manner. Int J Biol Sci. (2021) 17:2294–307. 10.7150/ijbs.57915 34239356 PMC8241722

[80] Huang R Hao C Wang D Zhao Q Li C Wang C. Spp1 derived from silica-exposed macrophage exosomes triggers fibroblast transdifferentiation. Toxicol Appl Pharmacol. (2021) 422:115559. 10.1016/j.taap.2021.115559 33961903

[81] Wang D Hao C Zhang L Zhang J Liu S Li Y. Exosomal Mir-125a-5p derived from silica-exposed macrophages induces fibroblast transdifferentiation. Ecotoxicol Environ Saf. (2020) 192:110253. 10.1016/j.ecoenv.2020.110253 32059163

[82] Qin X Lin X Liu L Li Y Li X Deng Z. Macrophage-derived exosomes mediate silica-induced pulmonary fibrosis by activating fibroblast in an endoplasmic reticulum stress-dependent manner. J Cell Mol Med. (2021) 25:4466–77. 10.1111/jcmm.16524 33834616 PMC8093963

[83] Li N Shi F Wang X Yang P Sun K Zhang L. Silica dust exposure induces pulmonary fibrosis through autophagy signaling. Environ Toxicol. (2021) 36:1269–77. 10.1002/tox.23124 33720480

[84] Li SX Li C Pang XR Zhang J Yu GC Yeo AJ. Metformin attenuates silica-induced pulmonary fibrosis by activating autophagy via the ampk-mtor signaling pathway. Front Pharmacol. (2021) 12:719589. 10.3389/fphar.2021.719589 34434111 PMC8381252

[85] Li W Xie L Ma J Cheng M Fan L Xu Y. Gas6 or Mer deficiency ameliorates silica-induced autophagosomes accumulation in mice lung. Toxicol Lett. (2021) 337:28–37. 10.1016/j.toxlet.2020.11.013 33232774

[86] Mizushima N Komatsu M. Autophagy: renovation of cells and tissues. Cell. (2011) 147:728–41. 10.1016/j.cell.2011.10.026 22078875

[87] Zhao H Wang Y Qiu T Liu W Yao P. Autophagy, an important therapeutic target for pulmonary fibrosis diseases. Clin Chim Acta. (2020) 502:139–47. 10.1016/j.cca.2019.12.016 31877297

[88] Tan S Chen S. Macrophage autophagy and silicosis: current perspective and latest insights. Int J Mol Sci. (2021) 22:453. 10.3390/ijms22010453 33466366 PMC7795780

[89] Jiang R Han L Gao Q Chao J. ZC3H4 mediates silica-induced EndoMT via ER stress and autophagy. Environ Toxicol Pharmacol. (2021) 84:103605. 10.1016/j.etap.2021.103605 33545378

[90] Liu Y Zhang X Wang J Yang F Luo W Huang J. ZC3H4 regulates infiltrating monocytes, attenuating pulmonary fibrosis through IL-10. Respir Res. (2022) 23:204. 10.1186/s12931-022-02134-2 35962397 PMC9375388

[91] Yang P Song R Li N Sun K Shi F Liu H. Silica dust exposure induces autophagy in alveolar macrophages through switching beclin1 affinity from BCL-2 to PIK3C3. Environ Toxicol. (2020) 35:758–67. 10.1002/tox.22910 32061152

[92] Qian Q Cao X Wang B Qu Y Qian Q Sun Z. TNF-Alpha-TNFR signal pathway inhibits autophagy and promotes apoptosis of alveolar macrophages in coal worker's pneumoconiosis. J Cell Physiol. (2019) 234:5953–63. 10.1002/jcp.27061 30467847

[93] Borges VM Lopes MF Falcao H Leite-Junior JH Rocco PR Davidson WF. Apoptosis underlies immunopathogenic mechanisms in acute silicosis. Am J Respir Cell Mol Biol. (2002) 27:78–84. 10.1165/ajrcmb.27.1.4717 12091249

[94] Borges VM Falcao H Leite-Junior JH Alvim L Teixeira GP Russo M. Fas ligand triggers pulmonary silicosis. J Exp Med. (2001) 194:155–64. 10.1084/jem.194.2.155 11457890 PMC2193452

[95] Santucci R Sinibaldi F Cozza P Polticelli F Fiorucci L. Cytochrome C: an extreme multifunctional protein with a key role in cell fate. Int J Biol Macromol. (2019) 136:1237–46. 10.1016/j.ijbiomac.2019.06.180 31252007

[96] Fazzi F Njah J Di Giuseppe M Winnica DE Go K Sala E. TNFR1/PHOX interaction and TNFR1 mitochondrial translocation thwart silica-induced pulmonary fibrosis. J Immunol. (2014) 192:3837–46. 10.4049/jimmunol.1103516 24623132 PMC3977215

[97] Li L Xu B Li CR Zhang MM Wu SJ Dang WJ. Anti-proliferation and apoptosis-inducing effects of sodium aescinate on retinoblastoma Y79 cells. Int J Ophthalmol. (2020) 13:1546–53. 10.18240/ijo.2020.10.06 33078103 PMC7511392

[98] Wang L Bowman L Lu Y Rojanasakul Y Mercer RR Castranova V. Essential role of P53 in silica-induced apoptosis. Am J Physiol Lung Cell Mol Physiol. (2005) 288:L488–96. 10.1152/ajplung.00123.2003 15557088

[99] Bhandary YP Shetty SK Marudamuthu AS Fu J Pinson BM Levin J. Role of P53-fibrinolytic system cross-talk in the regulation of quartz-induced lung injury. Toxicol Appl Pharmacol. (2015) 283:92–8. 10.1016/j.taap.2015.01.007 25596429

[100] Di Giuseppe M Gambelli F Hoyle GW Lungarella G Studer SM Richards T. Systemic inhibition of Nf-Kappab activation protects from silicosis. PLoS ONE. (2009) 4:e5689. 10.1371/journal.pone.0005689 19479048 PMC2682759

[101] Zhang L Xu D Li Q Yang Y Xu H Wei Z. N-Acetyl-Seryl-Aspartyl-Lysyl-Proline (Ac-Sdkp) attenuates silicotic fibrosis by suppressing apoptosis of alveolar type II epithelial cells via mediation of endoplasmic reticulum stress. Toxicol Appl Pharmacol. (2018) 350:1–10. 10.1016/j.taap.2018.04.025 29684394

[102] Huang Y Xu W Zhou R. NLRP3 inflammasome activation and cell death. Cell Mol Immunol. (2021) 18:2114–27. 10.1038/s41423-021-00740-6 34321623 PMC8429580

[103] Dostert C Petrilli V Van Bruggen R Steele C Mossman BT Tschopp J. Innate immune activation through NALP3 inflammasome sensing of asbestos and silica. Science. (2008) 320:674–7. 10.1126/science.1156995 18403674 PMC2396588

[104] Chen S Han B Geng X Li P Lavin MF Yeo AJ. Microcrystalline silica particles induce inflammatory response via pyroptosis in primary human respiratory epithelial cells. Environ Toxicol. (2022) 37:385–400. 10.1002/tox.23405 34766707

[105] Kang L Dai J Wang Y Shi P Zou Y Pei J. Blocking caspase-1/Gsdmd and caspase-3/-8/Gsdme pyroptotic pathways rescues silicosis in mice. PLoS Genet. (2022) 18:e1010515. 10.1371/journal.pgen.1010515 36459518 PMC9718385

[106] Zhang Q Ban J Chang S Qu H Chen J Liu F. The aggravate role of exosomal circrna11:120406118|12040782 on macrophage pyroptosis through mir-30b-5p/Nlrp3 axis in silica-induced lung fibrosis. Int Immunopharmacol. (2023) 114:109476. 10.1016/j.intimp.2022.109476 36450208

[107] Bhattacharjee P Paul S Bhattacharjee P. Risk of occupational exposure to asbestos, silicon and arsenic on pulmonary disorders: understanding the genetic-epigenetic interplay and future prospects. Environ Res. (2016) 147:425–34. 10.1016/j.envres.2016.02.038 26966890

[108] Zhang Y Gu P Xie Y Fan L You X Yang S. Insights into the mechanism underlying crystalline silica-induced pulmonary fibrosis via transcriptome-wide m(6)a methylation profile. Ecotoxicol Environ Saf. (2022) 247:114215. 10.1016/j.ecoenv.2022.114215 36306621

[109] Li Z Xue J Yan S Chen P Chen L. Association between tumor necrosis factor-alpha 308g/a gene polymorphism and silicosis susceptibility: a meta-analysis. PLoS ONE. (2013) 8:e76614. 10.1371/journal.pone.0076614 24124578 PMC3790741

[110] Zhang M Peng LL Ji XL Yang HB Zha RS Gui GP. Tumor necrosis factor gene polymorphisms are associated with silicosis: a systemic review and meta-analysis. Biosci Rep. (2019) 39:BSR20181896. 10.1042/BSR20181896 30643011 PMC6361771

[111] Fan Y Zheng C Wu N Li Y Huang X Ye Q. Telomerase gene variants and telomere shortening in patients with silicosis or asbestosis. Occup Environ Med. (2020) 15:107046. 10.1136/oemed-2020-107046 33323453

[112] Chu M Wu S Wang W Yu Y Zhang M Sang L. Functional variant of the carboxypeptidase m (CPM) gene may affect silica-related pneumoconiosis susceptibility by its expression: a multistage case-control study. Occup Environ Med. (2019) 76:169–74. 10.1136/oemed-2018-105545 30674606 PMC6581105

[113] Peng Z Duan M Zhao K Tang Y Liang F. Rab20 deficiency promotes the development of silicosis via NLRP3 inflammasome. Front Immunol. (2022) 13:967299. 10.3389/fimmu.2022.967299 36131930 PMC9484360

[114] Wang D Zhou M Liu Y Ma J Yang M Shi T. Comparison of risk of silicosis in metal mines and pottery factories: a 44-year cohort study. Chest. (2020) 158:1050–9. 10.1016/j.chest.2020.03.054 32298729

[115] Chen S Tan S Yang S Chen G Zhu L Sun Z. Nicotine induces apoptosis through exacerbation of blocked alveolar macrophage autophagic degradation in silicosis. Toxicol Lett. (2020) 334:94–101. 10.1016/j.toxlet.2020.09.019 33010382

[116] Chen S Yuan J Yao S Jin Y Chen G Tian W. Lipopolysaccharides may aggravate apoptosis through accumulation of autophagosomes in alveolar macrophages of human silicosis. Autophagy. (2015) 11:2346–57. 10.1080/15548627.2015.1109765 26553601 PMC4835201

[117] Cheng Z Zhang Y Zhao R Zhou Y Dong Y Qiu A. A novel circrna-snp may increase susceptibility to silicosis. Ecotoxicol Environ Saf. (2022) 242:113855. 10.1016/j.ecoenv.2022.113855 35835075

[118] Michaud G Reddy C Ernst A. Whole-lung lavage for pulmonary alveolar proteinosis. Chest. (2009) 136:1678–81. 10.1378/chest.09-2295 19995769

[119] Yang M Li B Wang B Li L Ji Y Zhou Y. Lung injury induced by different negative suction pressure in patients with pneumoconiosis undergoing whole lung lavage. BMC Pulm Med. (2022) 22:152. 10.1186/s12890-022-01952-w 35459122 PMC9034602

[120] Young KA Dilling DF. The future of lung transplantation. Chest. (2019) 155:465–73. 10.1016/j.chest.2018.08.1036 30171860 PMC6435913

[121] Yuan J Li P Pan H Xu Q Xu T Li Y. mir-770-5p inhibits the activation of pulmonary fibroblasts and silica-induced pulmonary fibrosis through targeting TGFBR1. Ecotoxicol Environ Saf. (2021) 220:112372. 10.1016/j.ecoenv.2021.112372 34082245

[122] Niu Z Wang L Qin X Ye Z Xie B Hu Y. Macrophage derived mir-7219-3p-containing exosomes mediate fibroblast trans-differentiation by targeting spry1 in silicosis. Toxicology. (2022) 479:153310. 10.1016/j.tox.2022.153310 36075289

[123] Tian Y Xia J Yang G Li C Qi Y Dai K. A2ar inhibits fibrosis and the emt process in silicosis by regulating Wnt/β-catenin pathway. Ecotoxicol Environ Saf. (2023) 249:114410. 10.1016/j.ecoenv.2022.114410 36516619

[124] Xie N Tan Z Banerjee S Cui H Ge J Liu RM. Glycolytic reprogramming in myofibroblast differentiation and lung fibrosis. Am J Respir Crit Care Med. (2015) 192:1462–74. 10.1164/rccm.201504-0780OC 26284610 PMC4731722

[125] Mao N Yang H Yin J Li Y Jin F Li T. Glycolytic reprogramming in silica-induced lung macrophages and silicosis reversed by ac-sdkp treatment. Int J Mol Sci. (2021) 22:10063. 10.3390/ijms221810063 34576239 PMC8465686

[126] Zhang J Cui J Li X Hao X Guo L Wang H. Increased secretion of VEGF-C from SiO(2)-induced pulmonary macrophages promotes lymphangiogenesis through the SRC/ENOS pathway in silicosis. Ecotoxicol Environ Saf. (2021) 218:112257. 10.1016/j.ecoenv.2021.112257 33933809

[127] Yu J Mao L Guan L Zhang Y Zhao J. Ginsenoside Rg1 enhances lymphatic transport of intrapulmonary silica via VEGF-C/VEGFR-3 signaling in silicotic rats. Biochem Biophys Res Commun. (2016) 472:182–8. 10.1016/j.bbrc.2016.02.091 26920056

[128] Han R Ji X Rong R Li Y Yao W Yuan J. Mir-449a regulates autophagy to inhibit silica-induced pulmonary fibrosis through targeting Bcl2. J Mol Med. (2016) 94:1267–79. 10.1007/s00109-016-1441-0 27351886

[129] Qian Q Ma Q Wang B Qian Q Zhao C Feng F. Microrna-205-5p targets E2F1 to promote autophagy and inhibit pulmonary fibrosis in silicosis through impairing Skp2-mediated beclin1 ubiquitination. J Cell Mol Med. (2021) 25:9214–27. 10.1111/jcmm.16825 34428336 PMC8500965

[130] Sun J Li Q Lian X Zhu Z Chen X Pei W. Microrna-29b mediates lung mesenchymal-epithelial transition and prevents lung fibrosis in the silicosis model. Mol Ther Nucleic Acids. (2019) 14:20–31. 10.1016/j.omtn.2018.10.017 30529807 PMC6282658

[131] Gao X Xu D Li S Wei Z Li S Cai W. Pulmonary silicosis alters microrna expression in rat lung and mir-411-3p exerts anti-fibrotic effects by inhibiting MRTF-A/SRF signaling. Mol Ther Nucleic Acids. (2020) 20:851–65. 10.1016/j.omtn.2020.05.005 32464548 PMC7256439

[132] Xu T Yan W Wu Q Xu Q Yuan J Li Y. Mir-326 Inhibits inflammation and promotes autophagy in silica-induced pulmonary fibrosis through targeting TNFSF14 and PTBP1. Chem Res Toxicol. (2019) 32:2192–203. 10.1021/acs.chemrestox.9b00194 31642316

[133] Wei Z Xu H Zhang Y Yi X Yang X Chen Y. Rho Gdp dissociation inhibitor alpha silencing attenuates silicosis by inhibiting RHOA/RHO kinase signalling. Exp Cell Res. (2019) 380:131–40. 10.1016/j.yexcr.2019.04.026 31029634

[134] Chen Y Song M Li Z Hou L Zhang H Zhang Z. Fcepsilonri deficiency alleviates silica-induced pulmonary inflammation and fibrosis. Ecotoxicol Environ Saf. (2022) 244:114043. 10.1016/j.ecoenv.2022.114043 36087468

[135] Liu H Cheng Y Yang J Wang W Fang S Zhang W. Bbc3 in macrophages promoted pulmonary fibrosis development through inducing autophagy during silicosis. Cell Death Dis. (2017) 8:e2657. 10.1038/cddis.2017.78 28277537 PMC5386570

[136] He R Yuan X Lv X Liu Q Tao L Meng J. Caveolin-1 negatively regulates inflammation and fibrosis in silicosis. J Cell Mol Med. (2022) 26:99–107. 10.1111/jcmm.17045 34889029 PMC8742238

[137] Goodman GB Kaplan PD Stachura I Castranova V Pailes WH Lapp NL. Acute silicosis responding to corticosteroid therapy. Chest. (1992) 101:366–70. 10.1378/chest.101.2.366 1735256

[138] Antonini JM McCloud CM Reasor MJ. Acute silica toxicity: attenuation by amiodarone-induced pulmonary phospholipidosis. Environ Health Perspect. (1994) 102:372–8. 10.1289/ehp.94102372 7925177 PMC1566962

[139] Podrid PJ. Amiodarone: reevaluation of an old drug. Ann Intern Med. (1995) 122:689–700. 10.7326/0003-4819-122-9-199505010-00008 7702232

[140] Li N Wu K Feng F Wang L Zhou X Wang W. Astragaloside IV alleviates silica-induced pulmonary fibrosis via inactivation of the TGF-Beta1/Smad2/3 signaling pathway. Int J Mol Med. (2021) 47:4849. 10.3892/ijmm.2021.4849 33448318 PMC7834968

[141] Zhang Y Li C Li S Lu Y Du S Zeng X. Dihydrotanshinone I alleviates crystalline silica-induced pulmonary inflammation by regulation of the TH immune response and inhibition of Stat1/Stat3. Mediators Inflamm. (2019) 2019:3427053. 10.1155/2019/3427053 31379467 PMC6652093

[142] Kumari S Singh R. Protective effects of intranasal curcumin on silica-induced lung damage. Cytokine. (2022) 157:155949. 10.1016/j.cyto.2022.155949 35764024

[143] Li C Lu Y Du S Li S Zhang Y Liu F. Dioscin exerts protective effects against crystalline silica-induced pulmonary fibrosis in mice. Theranostics. (2017) 7:4255–75. 10.7150/thno.20270 29158824 PMC5695011

[144] Du S Li C Lu Y Lei X Zhang Y Li S. Dioscin alleviates crystalline silica-induced pulmonary inflammation and fibrosis through promoting alveolar macrophage autophagy. Theranostics. (2019) 9:1878–92. 10.7150/thno.29682 31037145 PMC6485284

[145] Yuan L Sun Y Zhou N Wu W Zheng W Wang Y. Dihydroquercetin attenuates silica-induced pulmonary fibrosis by inhibiting ferroptosis signaling pathway. Front Pharmacol. (2022) 13:845600. 10.3389/fphar.2022.845600 35645837 PMC9133504

[146] Cao ZJ Liu Y Zhang Z Yang PR Li ZG Song MY. Pirfenidone ameliorates silica-induced lung inflammation and fibrosis in mice by inhibiting the secretion of interleukin-17a. Acta Pharmacol Sin. (2022) 43:908–18. 10.1038/s41401-021-00706-4 34316030 PMC8976043

[147] Tang Q Xing C Li M Jia Q Bo C Zhang Z. Pirfenidone ameliorates pulmonary inflammation and fibrosis in a rat silicosis model by inhibiting macrophage polarization and Jak2/Stat3 signaling pathways. Ecotoxicol Environ Saf. (2022) 244:114066. 10.1016/j.ecoenv.2022.114066 36108436

[148] Guo J Yang Z Jia Q Bo C Shao H Zhang Z. Pirfenidone inhibits epithelial-mesenchymal transition and pulmonary fibrosis in the rat silicosis model. Toxicol Lett. (2019) 300:59–66. 10.1016/j.toxlet.2018.10.019 30394303

[149] Wang M Zhang Z Liu J Song M Zhang T Chen Y. Gefitinib and fostamatinib target EGFR and SYK to attenuate silicosis: a multi-omics study with drug exploration. Signal Transduct Target Ther. (2022) 7:157. 10.1038/s41392-022-00959-3 35551173 PMC9098425

[150] Helal MG Said E. Carvedilol attenuates experimentally induced silicosis in rats via modulation of P-Akt/Mtor/Tgfbeta1 signaling. Int Immunopharmacol. (2019) 70:47–55. 10.1016/j.intimp.2019.02.011 30785090

[151] Song MY Wang JX Sun YL Han ZF Zhou YT Liu Y. Tetrandrine alleviates silicosis by inhibiting canonical and non-canonical NLRP3 inflammasome activation in lung macrophages. Acta Pharmacol Sin. (2022) 43:1274–84. 10.1038/s41401-021-00693-6 34417574 PMC9061833

[152] Chen S Tang K Hu P Tan S Yang S Yang C. Atractylenolide III alleviates the apoptosis through inhibition of autophagy by the mtor-dependent pathway in alveolar macrophages of human silicosis. Mol Cell Biochem. (2021) 476:809–18. 10.1007/s11010-020-03946-w 33078341

[153] Feng F Cheng P Zhang H Li N Qi Y Wang H. The protective role of tanshinone iia in silicosis rat model via Tgf-beta1/Smad signaling suppression, NOX4 inhibition and Nrf2/Are signaling activation. Drug Des Devel Ther. (2019) 13:4275–90. 10.2147/DDDT.S230572 31908414 PMC6930391

[154] Niu Z Lin J Hao C Xu X Wang C Dai K. Glycyrrhizic acid attenuates pulmonary fibrosis of silicosis by inhibiting the interaction between Hmgb1 and Brg1 through Pi3k/Akt/Mtor pathway. Int J Environ Res Public Health. (2022) 19:8743. 10.3390/ijerph19148743 35886594 PMC9317839

[155] Silva LHA Silva MC Vieira JB Lima ECD Silva RC Weiss DJ. Magnetic targeting increases mesenchymal stromal cell retention in lungs and enhances beneficial effects on pulmonary damage in experimental silicosis. Stem Cells Transl Med. (2020) 9:1244–56. 10.1002/sctm.20-0004 32538526 PMC7519769

[156] Chen S Cui G Peng C Lavin MF Sun X Zhang E. Transplantation of adipose-derived mesenchymal stem cells attenuates pulmonary fibrosis of silicosis via anti-inflammatory and anti-apoptosis effects in rats. Stem Cell Res Ther. (2018) 9:110. 10.1186/s13287-018-0846-9 29673394 PMC5909257

[157] de Oliveira HD de Melo EBB Silva JD Kitoko JZ Gutfilen B Barboza T. Therapeutic effects of bone marrow-derived mononuclear cells from healthy or silicotic donors on recipient silicosis mice. Stem Cell Res Ther. (2017) 8:259. 10.1186/s13287-017-0699-7 29126438 PMC5681761

[158] Xu C Zhao J Li Q Hou L Wang Y Li S. Exosomes derived from three-dimensional cultured human umbilical cord mesenchymal stem cells ameliorate pulmonary fibrosis in a mouse silicosis model. Stem Cell Res Ther. (2020) 11:503. 10.1186/s13287-020-02023-9 33239075 PMC7687745

[159] Xu C Hou L Zhao J Wang Y Jiang F Jiang Q. Exosomal Let-7i-5p from three-dimensional cultured human umbilical cord mesenchymal stem cells inhibits fibroblast activation in silicosis through targeting Tgfbr1. Ecotoxicol Environ Saf. (2022) 233:113302. 10.1016/j.ecoenv.2022.113302 35189518

[160] Guo H Su Y Deng F. Effects of mesenchymal stromal cell-derived extracellular vesicles in lung diseases: current status and future perspectives. Stem Cell Rev Rep. (2021) 17:440–58. 10.1007/s12015-020-10085-8 33211245 PMC7675022

[161] Yang YM Fukui M Wang Z Miao F Karriker MJ Seki E. Interventional potential of recombinant feline hepatocyte growth factor in a mouse model of non-alcoholic steatohepatitis. Front Endocrinol. (2018) 9:378. 10.3389/fendo.2018.00378 30083132 PMC6064873

[162] Panganiban RA Day RM. Hepatocyte growth factor in lung repair and pulmonary fibrosis. Acta Pharmacol Sin. (2011) 32:12–20. 10.1038/aps.2010.90 21131996 PMC4003323

[163] Duan H Gao P Cheng X Lu Y Hu C Zhu X. A nanoparticle delivery of plasmid encoding hepatocyte growth factor for gene therapy of silicosis in mice. J Pharm Pharm Sci. (2021) 24:488–98. 10.18433/jpps32218 34644525

[164] Liu WW Wang HX Yu W Bi XY Chen JY Chen LZ. Treatment of silicosis with hepatocyte growth factor-modified autologous bone marrow stromal cells: a non-randomized study with follow-up. Genet Mol Res. (2015) 14:10672–81. 10.4238/2015.September.9.7 26400297

[165] Ibarra-Sánchez L Gámez-Méndez A Martínez-Ruiz M Nájera-Martínez EF Morales-Flores BA Melchor-Martínez EM. Nanostructures for drug delivery in respiratory diseases therapeutics: revision of current trends and its comparative analysis. J Drug Deliv Sci Technol. (2022) 70:103219. 10.1016/j.jddst.2022.103219 35280919 PMC8896872

[166] Liu S Chen D Li X Guan M Zhou Y Li L. Fullerene nanoparticles: a promising candidate for the alleviation of silicosis-associated pulmonary inflammation. Nanoscale. (2020) 12:17470–9. 10.1039/D0NR04401F 32808001

[167] Tan S Chen S. The mechanism and effect of autophagy, apoptosis, and pyroptosis on the progression of silicosis. Int J Mol Sci. (2021) 22:8110. 10.3390/ijms22158110 34360876 PMC8348676

[168] Soldner F Jaenisch R. Stem cells, genome editing, and the path to translational medicine. Cell. (2018) 175:615–32. 10.1016/j.cell.2018.09.010 30340033 PMC6461399

[169] Karczewski KJ Snyder MP. Integrative omics for health and disease. Nat Rev Genet. (2018) 19:299–310. 10.1038/nrg.2018.4 29479082 PMC5990367

[170] Samsonraj RM Raghunath M Nurcombe V Hui JH van Wijnen AJ Cool SM. Concise review: multifaceted characterization of human mesenchymal stem cells for use in regenerative medicine. Stem Cells Transl Med. (2017) 6:2173–85. 10.1002/sctm.17-0129 29076267 PMC5702523

